# Deep learning to detect left ventricular structural abnormalities in chest X-rays

**DOI:** 10.1093/eurheartj/ehad782

**Published:** 2024-03-20

**Authors:** Shreyas Bhave, Victor Rodriguez, Timothy Poterucha, Simukayi Mutasa, Dwight Aberle, Kathleen M Capaccione, Yibo Chen, Belinda Dsouza, Shifali Dumeer, Jonathan Goldstein, Aaron Hodes, Jay Leb, Matthew Lungren, Mitchell Miller, David Monoky, Benjamin Navot, Kapil Wattamwar, Anoop Wattamwar, Kevin Clerkin, David Ouyang, Euan Ashley, Veli K Topkara, Mathew Maurer, Andrew J Einstein, Nir Uriel, Shunichi Homma, Allan Schwartz, Diego Jaramillo, Adler J Perotte, Pierre Elias

**Affiliations:** Division of Cardiology and Department of Biomedical Informatics, Columbia University Irving Medical Center, 622 West 168th Street, PH20, NewYork, NY 10032, USA; Division of Cardiology and Department of Biomedical Informatics, Columbia University Irving Medical Center, 622 West 168th Street, PH20, NewYork, NY 10032, USA; Seymour, Paul, and Gloria Milstein Division of Cardiology, Department of Medicine, Columbia University Irving Medical Center, NewYork-Presbyterian Hospital, 630 West 168th Street, NewYork, NY 10032, USA; Department of Radiology, Columbia University Irving Medical Center, NewYork, NY, USA; Department of Radiology, Columbia University Irving Medical Center, NewYork, NY, USA; Department of Radiology, Columbia University Irving Medical Center, NewYork, NY, USA; Inova Fairfax Hospital Imaging Center, Inova Fairfax Medical Campus, Falls Church, VA, USA; Department of Radiology, Columbia University Irving Medical Center, NewYork, NY, USA; Department of Radiology, Columbia University Irving Medical Center, NewYork, NY, USA; Department of Radiology, Columbia University Irving Medical Center, NewYork, NY, USA; Hackensack Radiology Group, Hackensack Meridian School of Medicine, Nutley, NJ, USA; Department of Radiology, Columbia University Irving Medical Center, NewYork, NY, USA; Department of Radiology, University of California, SanFrancisco, CA, USA; Hackensack Radiology Group, Hackensack Meridian School of Medicine, Nutley, NJ, USA; Hackensack Radiology Group, Hackensack Meridian School of Medicine, Nutley, NJ, USA; Department of Radiology, Columbia University Irving Medical Center, NewYork, NY, USA; Division of Vascular and Interventional Radiology, Department of Radiology, Montefiore Medical Center, Bronx, NY, USA; Hackensack Radiology Group, Hackensack Meridian School of Medicine, Nutley, NJ, USA; Seymour, Paul, and Gloria Milstein Division of Cardiology, Department of Medicine, Columbia University Irving Medical Center, NewYork-Presbyterian Hospital, 630 West 168th Street, NewYork, NY 10032, USA; Smidt Heart Institute, Cedars-Sinai Medical Center, Los Angeles, CA, USA; Stanford Center for Inherited Cardiovascular Disease, Stanford University School of Medicine, Palo Alto, CA, USA; Seymour, Paul, and Gloria Milstein Division of Cardiology, Department of Medicine, Columbia University Irving Medical Center, NewYork-Presbyterian Hospital, 630 West 168th Street, NewYork, NY 10032, USA; Seymour, Paul, and Gloria Milstein Division of Cardiology, Department of Medicine, Columbia University Irving Medical Center, NewYork-Presbyterian Hospital, 630 West 168th Street, NewYork, NY 10032, USA; Seymour, Paul, and Gloria Milstein Division of Cardiology, Department of Medicine, Columbia University Irving Medical Center, NewYork-Presbyterian Hospital, 630 West 168th Street, NewYork, NY 10032, USA; Department of Radiology, Columbia University Irving Medical Center, NewYork, NY, USA; Seymour, Paul, and Gloria Milstein Division of Cardiology, Department of Medicine, Columbia University Irving Medical Center, NewYork-Presbyterian Hospital, 630 West 168th Street, NewYork, NY 10032, USA; Seymour, Paul, and Gloria Milstein Division of Cardiology, Department of Medicine, Columbia University Irving Medical Center, NewYork-Presbyterian Hospital, 630 West 168th Street, NewYork, NY 10032, USA; Seymour, Paul, and Gloria Milstein Division of Cardiology, Department of Medicine, Columbia University Irving Medical Center, NewYork-Presbyterian Hospital, 630 West 168th Street, NewYork, NY 10032, USA; Department of Radiology, Columbia University Irving Medical Center, NewYork, NY, USA; Division of Cardiology and Department of Biomedical Informatics, Columbia University Irving Medical Center, 622 West 168th Street, PH20, NewYork, NY 10032, USA; Division of Cardiology and Department of Biomedical Informatics, Columbia University Irving Medical Center, 622 West 168th Street, PH20, NewYork, NY 10032, USA; Seymour, Paul, and Gloria Milstein Division of Cardiology, Department of Medicine, Columbia University Irving Medical Center, NewYork-Presbyterian Hospital, 630 West 168th Street, NewYork, NY 10032, USA

**Keywords:** Deep learning, Chest X-rays, Early detection, Heart failure, Dilated left ventricle, Left ventricular hypertrophy

## Abstract

**Background and Aims:**

Early identification of cardiac structural abnormalities indicative of heart failure is crucial to improving patient outcomes. Chest X-rays (CXRs) are routinely conducted on a broad population of patients, presenting an opportunity to build scalable screening tools for structural abnormalities indicative of Stage B or worse heart failure with deep learning methods. In this study, a model was developed to identify severe left ventricular hypertrophy (SLVH) and dilated left ventricle (DLV) using CXRs.

**Methods:**

A total of 71 589 unique CXRs from 24 689 different patients completed within 1 year of echocardiograms were identified. Labels for SLVH, DLV, and a composite label indicating the presence of either were extracted from echocardiograms. A deep learning model was developed and evaluated using area under the receiver operating characteristic curve (AUROC). Performance was additionally validated on 8003 CXRs from an external site and compared against visual assessment by 15 board-certified radiologists.

**Results:**

The model yielded an AUROC of 0.79 (0.76–0.81) for SLVH, 0.80 (0.77–0.84) for DLV, and 0.80 (0.78–0.83) for the composite label, with similar performance on an external data set. The model outperformed all 15 individual radiologists for predicting the composite label and achieved a sensitivity of 71% vs. 66% against the consensus vote across all radiologists at a fixed specificity of 73%.

**Conclusions:**

Deep learning analysis of CXRs can accurately detect the presence of certain structural abnormalities and may be useful in early identification of patients with LV hypertrophy and dilation. As a resource to promote further innovation, 71 589 CXRs with adjoining echocardiographic labels have been made publicly available.

## Structured Graphical Abstract

**Structured Graphical Abstract:**
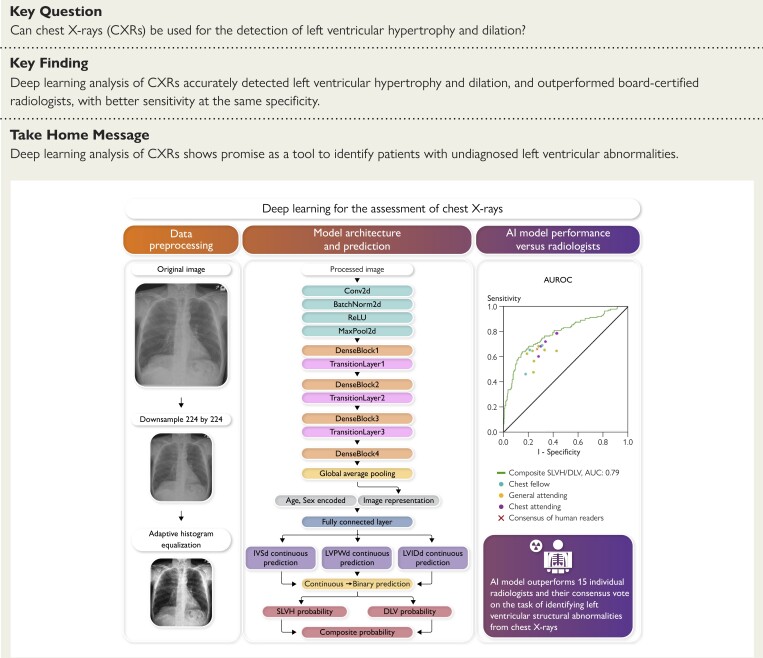
The deep learning model that was developed takes as input a pre-processed chest X-ray of dimension 224-by-224, age, and sex; the model outputs a probability for dilated left ventricle, severe left ventricular hypertrophy, and a composite label indicating the presence of either structural abnormality. The model outperforms all 15 board-certified radiologists in the task of detecting the presence of either abnormality (composite label). For a single point of comparison, we used the consensus vote amongst radiologists. The model achieves a sensitivity of 71% compared with the consensus vote sensitivity of 66% at a fixed specificity of 73%. Saliency maps demonstrate that at shallower layers in the network, the model is sensitive to the broader cardiac silhouette as well as structures in the left heart. AI, artificial intelligence; AUROC, area under the receiver operating characteristic curve; CXR, chest X-ray; DLV, dilated left ventricle; IVSd, interventricular septal thickness at end-diastole; LVIDd, left ventricular internal diameter at end-diastole; LVPWd, left ventricular posterior wall distance at end-diastole; SLVH, severe left ventricular hypertrophy.

**See the editorial comment for this article ‘A deep learning solution to detect left ventricular structural abnormalities with chest X-rays: towards trustworthy AI in cardiology’, by K. Lekadir, https://doi.org/10.1093/eurheartj/ehad775.**

## Introduction

Early identification of structural changes to the heart is critical to improving outcomes for patients with heart failure. Initial signs and symptoms of early-stage heart failure can be non-specific, often resulting in delays in diagnostic echocardiography.^1,2^ Millions of patients with left ventricular (LV) structural abnormalities remain undiagnosed and later diagnosis is associated with worse outcomes.^1–3^ Routinely diagnosing heart failure earlier, ideally when only structural abnormalities are present but patients are not yet symptomatic, remains an elusive but critical goal in cardiology.^4^ An accurate, broadly applicable, and cost-effective method of detecting LV structural abnormalities could lead to earlier diagnosis of heart failure and improved outcomes.

Echocardiography is the primary diagnostic study for LV structural abnormalities^5–8^ but is usually only performed on a narrow patient population with high pre-test probability.^9,10^ On the other hand, chest X-rays (CXRs) are relatively inexpensive and much more frequently performed on a broader population of patients.^11,12^ Recent work has shown that deep learning methods can effectively detect reduced ejection fraction,^13,14^ valvular heart disease,^15–17^ and LV hypertrophy^18^ from cardiac tests such as echocardiograms and electrocardiograms. However, there has been limited work on building methods for detecting cardiac pathology with CXRs. Identification of incidental cardiac abnormalities in patients undergoing a CXR may allow for earlier recognition and treatment of cardiac disease. In this study, we sought to use CXRs to detect two primary cardiac structural abnormalities indicative of heart failure: severe LV hypertrophy (SLVH) and dilated left ventricle (DLV).

Deep learning methods utilizing CXRs have lacked both variety and quality in labels for cardiac conditions. Prior models^19,20^ have primarily focused on detecting cardiomegaly, a non-specific term to indicate an abnormally enlarged heart. However, radiographic cardiomegaly is poorly predictive of cardiac disease, and there are no guideline-directed recommendations for further workup in its presence.^21,22^ We sought to determine whether more clinically actionable diagnoses of SLVH and DLV derived from echocardiograms could be detected using CXRs. In this study, we (i) developed a deep learning model to accurately identify SLVH and DLV using CXRs, (ii) assessed model performance when applied to a different hospital’s data, (iii) compared the model’s accuracy in detecting pathology vs. that of 15 radiologists, and (iv) applied saliency mapping to determine which parts of the CXRs the model were most sensitive to in making predictions. The development of an artificial intelligence (AI) model that can accurately detect LV structural abnormalities from inexpensive, prevalent exams like CXRs represents a novel solution to diagnosing heart failure earlier in the disease process.

## Methods

### Data sources and cohort construction

All patients who had both a CXR and an echocardiogram conducted at Columbia University Irving Medical Center (CUIMC) from January 2013 to August 2018 were identified. Chest X-rays in their full resolution were extracted in DICOM format and filtered to only include posteroanterior (PA) films. All portable anteroposterior films were excluded to prevent label leakage from the model potentially associating portable films with patients more likely to have cardiac pathology. Chest X-ray metadata were used to identify demographic information including age and sex. The echocardiograms were accessed through the Syngo Dynamics system (Siemens Healthineers, Malvern, PA, USA). For each echocardiogram, the following continuous measures were extracted from the parasternal long-axis view using our enterprise data warehouse that stores finalized reports: interventricular septal thickness at end-diastole (IVSd), LV internal diameter at end-diastole (LVIDd), and LV posterior wall distance at end-diastole (LVPWd).

After extraction, only CXRs for patients with at least one echocardiogram conducted within 12 months (i.e. before or after CXR) were retained in the final data set. In constructing the data set, our objective was to create pairs of CXRs and echocardiograms close enough in temporal proximity where signs of structural abnormality could plausibly be present on a CXR, given a positive indication via echocardiogram. The data set contains patients who may have heart failure at any stage (i.e. asymptomatic or symptomatic). While structural changes may take place over the course of 12 months, these changes are typically slow to progress. To assess the impact of choosing a different cut-off window for associating a CXR with an echocardiogram-derived label, we evaluated how drastically IVSd, LVIDd, LVPWd, and LV ejection fraction (LVEF) change between successive echocardiograms conducted on the same patient (Supplementary data online, *Figure S7*). It was determined that the magnitude of the difference in measurements was not significantly different when the temporal difference between successive echocardiograms was larger.

**Figure 1:**
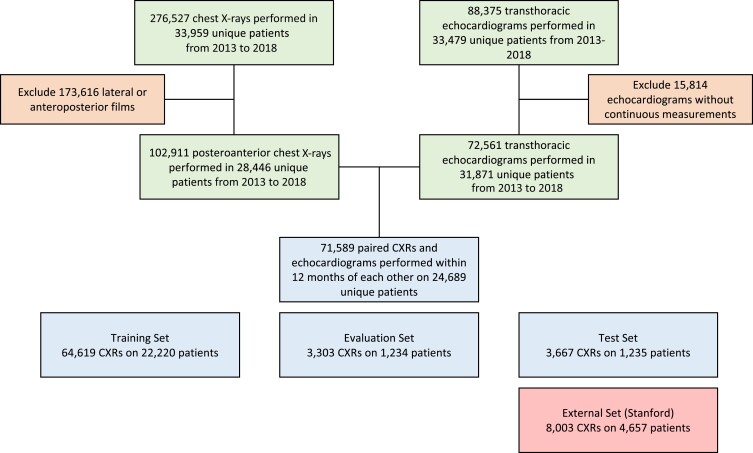
Cohort construction. Chest X-rays in their full resolution were extracted in DICOM format and filtered to only include posteroanterior frontal films. Echocardiograms were filtered to only those with all three continuous measurements of interest (interventricular septal thickness at end-diastole, left ventricular internal diameter at end-diastole, and left ventricular posterior wall distance at end-diastole) recorded. Only chest X-rays for patients with at least one echocardiogram conducted within 12 months were retained in the final data set. The final data set consisted of 71 589 unique chest X-rays conducted on 24 689 different patients. Each patient in the final cohort was randomly assigned to a training (90%), validation (5%), or test set (5%) to ensure that chest X-rays for a given patient were limited to a single partition. CXR, chest X-ray.

The final data set consisted of 71 589 unique CXRs conducted on 24 689 different patients (*Figure 1*). Using the echocardiographic measurements, gold-standard labels for IVSd, LVIDd, and LVPWd were assigned to each CXR. For any CXR with multiple echocardiograms conducted within 12 months, the maximum of each echocardiographic measurement across all studies was taken. A majority of CXR and echocardiogram pairs were conducted within 6 months of each other, indicating that continuous measurements recorded via echocardiogram are in close temporal proximity to the CXR (Supplementary data online, *Figures S5* and *S6*). Binary diagnosis labels for SLVH and DLV were derived by thresholding echocardiographic measurements in accordance with current guidelines.^8^ Men with IVSd > 1.5 cm or LVPWd > 1.5 cm and women with IVSd > 1.4 cm or LVPWd > 1.4 cm were identified as SLVH cases. Meanwhile, men with LVIDd > 5.9 cm and women with LVIDd > 5.3 cm were identified as DLV cases. A composite binary label was also constructed indicating the presence of either SLVH or DLV. We chose to focus on the modelling SLVH in lieu of mild and moderate LVH for two primary reasons: (i) we hypothesized that CXRs would not carry a rich enough signal for the detection of mild/moderate cases, and (ii) we wanted to ensure a fair comparison with radiologist assessments, since a pre-study survey of radiologists indicated that mild/moderate cases would not be easily indicated on a CXR if read by human experts.

Each patient in the final cohort was randomly assigned to a training (90%), validation (5%), or test set (5%) to ensure that CXRs for a given patient were limited to a single partition. As a resource to promote further innovation, we have made all CXRs with adjoining echocardiographic labels a publicly available data set.

To assess how well our model performed on data from another institution, we reported its performance on a data set from Stanford University Medical Center consisting of 8003 PA CXRs from 4657 patients completed between October 2002 and July 2017 where an echocardiogram was completed within 12 months. The data set was curated using a procedure identical to that used for building the CUIMC data set with the exception that echocardiographic data were abstracted from a Philips Xcelera system.

### Data pre-processing and model architecture

Chest X-rays were first cropped to a 1:1 aspect ratio and downsampled to a 224-by-224 pixel image using bicubic interpolation to ensure images were the same size. To improve the contrast of images, contrast-limited adaptive histogram equalization^23^ was applied to each image and Gaussian noise was added to each image in the training set to improve generalization performance. The DenseNet-121^24^ architecture was used as the backbone of the model as it has been shown to learn effective representations of CXRs using a series of convolutions and residual connections.^19,20,25^ The representation of the image at the last layer of the neural network along with sex and the continuous age of the patient at the time of the CXR was combined to produce a single data vector. Using this vector, the model produced estimates for the three continuous echocardiogram measurements: LVPWd, IVSd, and LVIDd. Based on these estimates, a probability for each of the binary labels (SLVH, DLV, and composite SLVH/DLV) was computed and used for assessing the evaluation metrics (*Structured Graphical Abstract*). Further details about the model architecture and optimization are included in the Supplementary data online, *Methods S1*.

### Model evaluation

We conducted three primary evaluations of the model: (i) internal site validation evaluating the trained model on a held-out test set, (ii) external validation on an independent test set obtained from a different institution, and (iii) comparison of model performance to that of 15 radiologists on a subset of sampled CXRs. We also assessed the performance of our model on key subpopulations: (i) a subset including only CXRs from the 12 months preceding the patient’s first echocardiogram to determine if performance was similar before a diagnosis of heart failure was made and (ii) a subset excluding patients with pacemakers (PMs), heart transplants (HTs), and lung transplants (LTs); the model may perform well in each of these populations by using information not related to cardiac anatomy (e.g. learning to positively classify patients when a PM present). The metrics we reported are area under the receiver operating characteristic curve (AUROC), area under the precision–recall curve, sensitivity, specificity, and positive predictive value (PPV). We used bootstrapping methods to construct 95% confidence intervals (CIs) around each statistic. Since multiple CXRs may be present for each patient, we also report the test set results with one CXR per patient (i.e. the average across resampling a single random CXR per patient). See Supplementary data online, *Methods S1* for more details.

### Chest X-rays prior to first echo analysis

For evaluating the model on a patient population more likely to be asymptomatic, we evaluated its performance on patients who had CXRs performed prior to their first recorded echocardiogram at CUIMC. Patients in this subpopulation are of interest because their pre-test probability for structural disease is lower since their providers have yet to order an echocardiogram. For the CUIMC test set, we isolated all CXRs conducted prior to a first echocardiogram and evaluated using the same performance statistics described previously.

### Exclusion of pacemakers and heart/lung transplant cases

Medical device implantation and organ transplants are common interventions in cases of late-stage heart failure. This patient population is enriched for structural heart problems, including SLVH and DLV. Deep learning models are prone to learning spurious confounders^26^ and exploiting them for prediction. This can cause failures^27^ when models are deployed in populations where such correlations may not exist. To ensure that the model was not using these attributes to infer patient status, we identified and excluded patients with PM, implantable cardioverter defibrillators (ICDs), or HTs/LTs. This was done by compiling a list of common phrases representing each concept and using regular expressions to identify their mentions within the diagnostic statements accompanying each CXR (Supplementary data online, *Table S1*). This identification method had 95% accuracy in a manual review of 200 randomly selected studies.

Thereby, we identified three categories of CXR based on the presence of (i) PM/ICD, (ii) HT, or (iii) LT. We constructed data subsets by excluding each category (and combinations thereof). For each subset, we generated new partitions, retrained, and re-evaluated to compare held-out performance against models trained using the full data set.

### Comparison with radiologists

To provide a comparison of model performance against experts, we recruited 15 board-certified radiologists. Ten were academic radiologists, with the remaining practicing in community hospitals. Five were chest sub-specialty attending physicians, three were completing chest fellowship, and the remaining were general radiology attending physicians. All regularly read CXRs as part of their practice; the average number of years of experience as radiologists was 11.4 years. A data set was constructed consisting of 204 images from the CUIMC test set and 204 images from the Stanford test set. We sampled 68 images each from CUIMC and Stanford (one-third of each sample), which were positive for the composite label, ensuring that SLVH and DLV were each present in at least half of the positives. The remaining images were randomly sampled from the set of CXRs, which were negative for the composite label.

A pre-study survey of radiologists found that cardiomegaly could represent one of four underlying pathologies: SLVH, DLV, dilated right ventricle, and large pericardial effusion. The latter two pathologies were present in <1% of the total data, and studies with either present were excluded in the head-to-head comparison to ensure the radiologist’s definition of cardiomegaly aligned with the model prediction. Each radiologist was given the full resolution CXRs to read and asked to label each for the presence of cardiomegaly as they would in routine clinical practice; see Supplementary data online, *Methods S1* for the exact prompt given to radiologists. The radiologists were informed that their interpretations would be compared with an AI model for accuracy of detecting SLVH or DLV. We compared the model’s performance on the same set of images against each individual radiologist and against the consensus vote across all radiologists.

### Saliency mapping

Class activation mapping^28^ (CAM) was used to visualize the regions within CXRs to which our model’s output was most sensitive. Class activation mapping allowed us to assess if the model was sensitive to components of a CXR, which could reasonably be relied upon to estimate the sizes of cardiac structures. Similarly, they allowed us to inspect the model’s sensitivity to PMs, LV assist devices, and other implanted medical devices that may have been informative confounders to the presence of SLVH and DLV. There are growing concerns that current state-of-the-art CAM approaches can lead to a false sense of assuredness that models are not attending to confounders. We therefore visualized layers at various depths using LayerCAM,^29^ a newer CAM algorithm developed specifically for this purpose. For further details about how these visualizations were generated, see the Supplementary data online, *Methods S1*.

## Results

We identified 71 589 unique CXRs conducted on 24 689 different patients completed within 1 year of an echocardiogram. The data set consisted of more females than males (57% female and 43% male) and a mean patient age of 62 years (*Table 1*). The label prevalences were as follows: 8.7% positive for SLVH, 6.0% positive for DLV, and 13.8% positive for the composite label. Using just age and sex, we fit logistic regression and gradient-boosted trees for each of the three binary labels. Age and sex alone were poor predictors for all three of the labels with an AUROC ranging from 0.51 to 0.59 (Supplementary data online, *Table S2*).

**Table 1:** Patient characteristics

	CUIMC train	CUIMC validation	CUIMC test	Stanford external
CXRs, *n*	64 619	3303	3667	8003
Patients, *n*	22 220	1234	1235	4657
Age, years	62.2 ± 16.2	62.3 ± 16.4	62.1 ± 16.2	59.0 ± 16.5
Age groups, years				
<59	25 493 (39.5)	1334 (40.4)	1428 (38.9)	3812 (47.6)
60–69	17 508 (27.1)	844 (25.6)	1032 (28.1)	2000 (25.0)
70–79	13 324 (20.6)	658 (19.9)	711 (19.4)	1336 (16.7)
80+	8294 (12.8)	467 (14.1)	496 (13.5)	855 (10.7)
Female sex	12 633 (56.9)	696 (56.4)	707 (57.2)	1820 (39.1)
Echo measures				
IVSd 2D	1.12 ± 0.27	1.12 ± 0.26	1.12 ± 0.23	1.17 ± 0.23
LVPWd 2D	1.07 ± 0.23	1.07 ± 0.22	1.07 ± 0.20	1.14 ± 0.21
LVd 2D	4.61 ± 0.68	4.65 ± 0.73	4.58 ± 0.60	5.01 ± 0.99
LVEF	56.2 ± 11.2	55.2 ± 11.9	56.6 ± 11.2	–
Labels				
SLVH	5621 (8.7)	301 (9.1)	269 (7.3)	826 (10.3)
DLV	3898 (6.0)	300 (9.1)	145 (4.0)	1368 (17.1)
Composite	8889 (13.8)	580 (17.6)	392 (10.7)	2016 (25.2)

Data are reported as mean ± SD or *n* (%). The only patient-level static characteristic is sex. All other statistics are summarized on a per CXR–echocardiogram pair basis (including age as it may change across different CXRs for the same patient).

### Model performance

**Figure 2:**
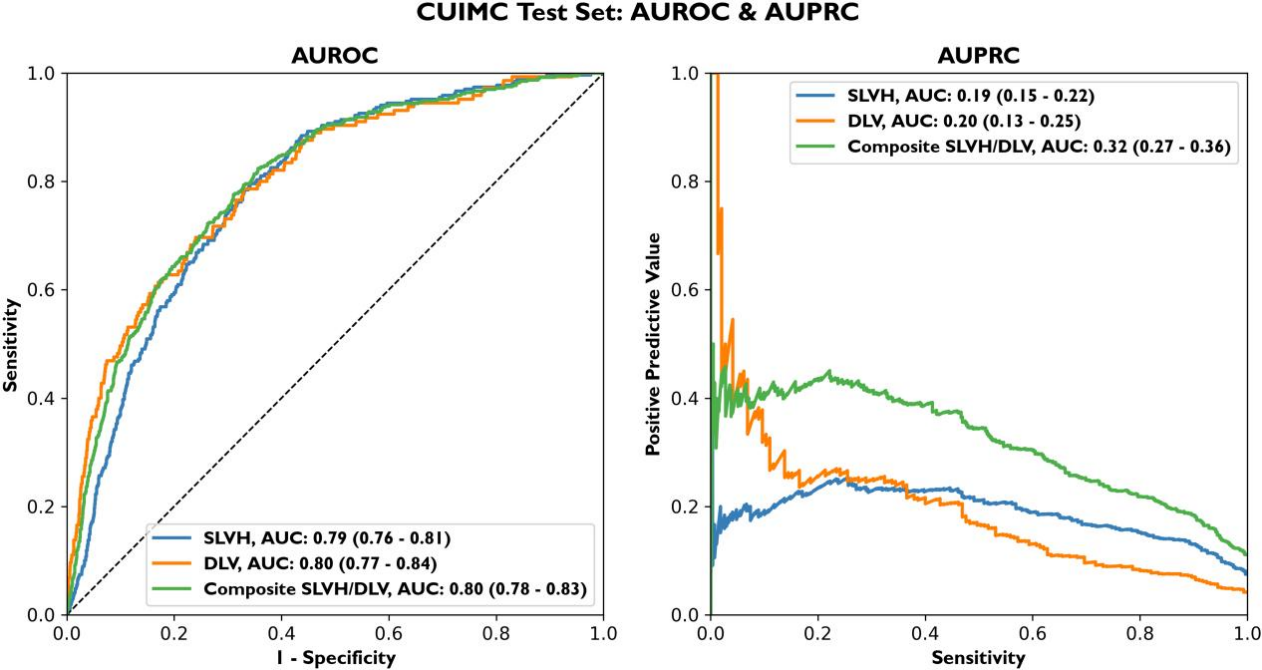
Columbia University Irving Medical Center test set area under the receiver operating characteristic curve/area under the precision–recall curve. The deep learning model fit on Columbia University Irving Medical Center data achieves an area under the receiver operating characteristic curve of 0.79 (0.76–0.81) for severe left ventricular hypertrophy, 0.80 (0.77–0.84) for dilated left ventricle, and 0.80 (0.78–0.83) for the composite label on the test set. The precision–recall curve shows the trade-off between positive predictive value (*y*-axis) and sensitivity (*x*-axis). For example, on the composite label, the model has a positive predictive value of 35% at a sensitivity of 50% and a specificity of 89%. Dilated left ventricle and severe left ventricular hypertrophy, by comparison, have lower area under the precision–recall curve values partly due to a lower baseline prevalence. CUIMC, Columbia University Irving Medical Center; AUROC, area under the receiver operating characteristic curve; AUPRC, area under the precision–recall curve; DLV, dilated left ventricle; SLVH, severe left ventricular hypertrophy.

The full model, which takes as input a single CXR, age, and sex, produces predicted probabilities for SLVH, DLV, and the composite label. This model yielded an AUROC of 0.79 (95% CI 0.76–0.81) for SLVH, 0.80 (95% CI 0.77–0.84) for DLV, and 0.80 (95% CI 0.78–0.83) for the composite label (*Figure 2*). Model performance was similar in an analysis restricted to a single randomly selected CXR per patient (Supplementary data online, *Table S3*). Performance was not significantly different when stratified by months between CXR and echocardiogram, suggesting that the choice to pair CXRs and echocardiograms as many as 12 months apart did not have a significant impact on label quality (Supplementary data online, *Figure S8*).

**Figure 3:**
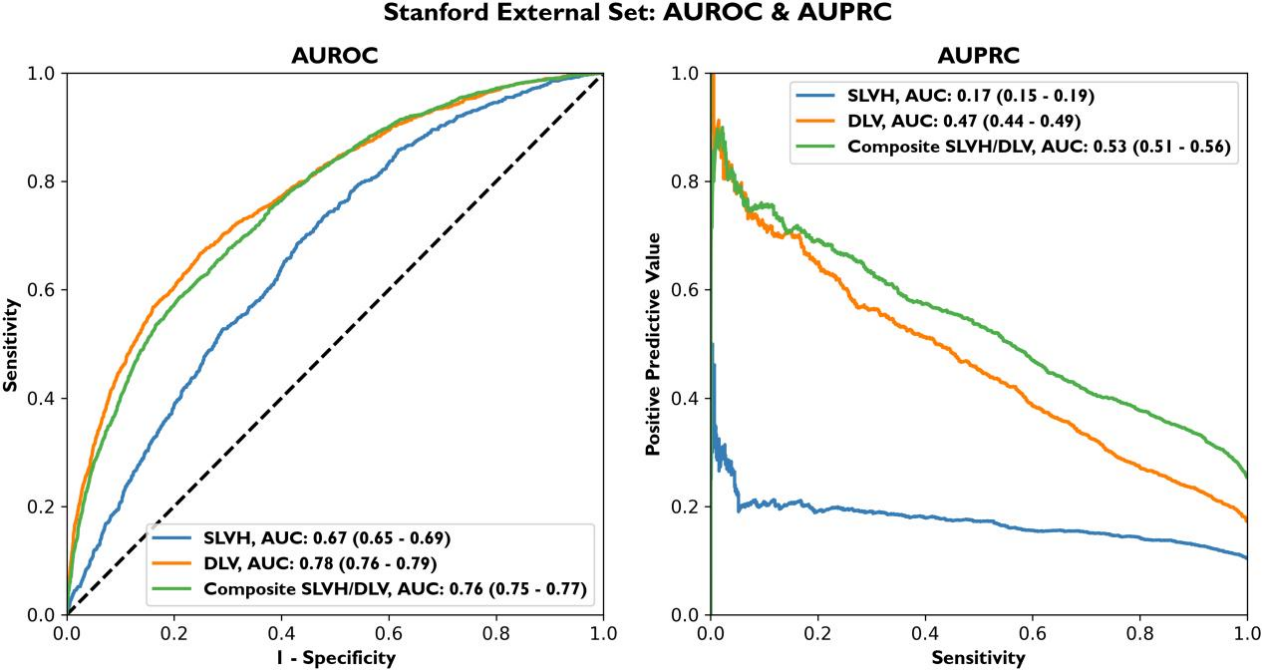
Stanford external set area under the receiver operating characteristic curve/area under the precision–recall curve. The external data set from Stanford University Medical center consisted of 8003 posteroanterior chest X-rays conducted on 4657 patients. The model’s area under the receiver operating characteristic curve on this external data set was 0.67 (95% confidence interval 0.65–0.69) for severe left ventricular hypertrophy, 0.78 (95% confidence interval 0.76–0.79) for dilated left ventricle, and 0.76 (95% confidence interval 0.75–0.77) for the composite label. The area under the precision–recall curve for the composite label is 0.53 (95% confidence interval 0.51–0.56) partly due to a higher prevalence of both severe left ventricular hypertrophy and dilated left ventricle. AUROC, area under the receiver operating characteristic curve; AUPRC, area under the precision–recall curve; DLV, dilated left ventricle; SLVH, severe left ventricular hypertrophy.

Using this trained model, we also evaluated our performance on a separate validation set from Stanford University (8003 CXRs on 4657 patients). In this data set, the AUROC was 0.67 (95% CI 0.65–0.69) for SLVH, 0.78 (95% CI 0.76–0.79) for DLV, and 0.76 (95% CI 0.75–0.77) for the composite label (*Figure 3* and Supplementary data online, *Table S3*). Performance suffered more significantly on the SLVH label as compared with DLV and the composite label. The label prevalences in this external data set were larger than those of Columbia’s data set (10.3% for SLVH, 17.1% for DLV, and 25.2% for the composite label), and the data set had a higher proportion of men than women (39% female and 61% male), though mean age was similar at 59 years (*Table 1*).

### Performance on chest X-rays done prior to first echocardiogram

The patient population used to train the model consisted of patients who may have already received an echocardiogram prior to a CXR. In a deployment scenario, the most impactful use case of the model would be to identify patients who have high risk for previously undiagnosed heart failure. To evaluate how the model performed on this key subpopulation, we isolated CXRs conducted prior to the first recorded echocardiogram for every patient in the test set. On this population, the model maintained an AUROC of 0.80 (95% CI 0.75–0.86) on the composite label (Supplementary data online, *Table S4*).

### Performance removing potential imaging confounders

We reported the performance characteristics (Supplementary data online, *Tables S5* and *S8*) of three models that were each re-trained by excluding different subpopulations: (i) excluding all PM and ICD, (ii) excluding all LT and HT patients, and (iii) excluding both (i) and (ii). The characteristics of each of these subpopulations are available in Supplementary data online, *Table S6*. There was no significant difference in composite label performance across all models, indicating stable model performance even in populations excluding these patient subtypes.

### Performance in demographic subpopulations

It is well-known that certain patient populations can have a higher baseline risk for heart failure. Black adults are at higher risk of heart failure compared with White adults.^30^ Similar trends have been noted in the case of ethnicity, where Hispanic adults are at a higher risk compared with non-Hispanics.^31^ We conducted an analysis of performance on four additional subpopulations across which risk is likely to be different: ethnicity, race, sex, and age (Supplementary data online, *Table S7*). We found that there is no significant difference in model performance on the composite label across non-Hispanics and Hispanics nor across Black and White patients (Supplementary data online, *Figure S4*). Performance was also similar across males and females, as well as across different age groups. This finding suggests that the model may perform well even within populations in which patients are at different baseline levels of risk.

### Comparison with radiologists

**Figure 4:**
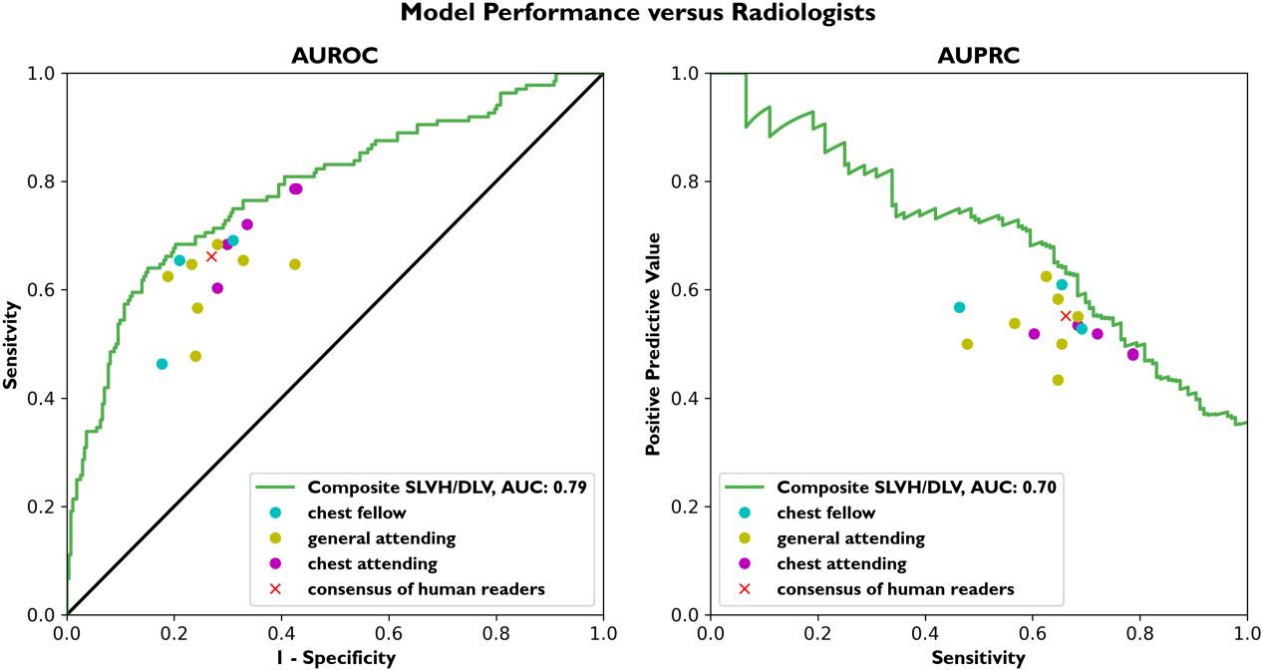
Model performance vs. radiologists. A separate data set of 408 chest X-rays sampled from Columbia University Irving Medical Center and Stanford data (204 from each) was used to compare model performance against radiologists. Each radiologist was given the full resolution chest X-rays to read and asked to label each for the presence of cardiomegaly as they would in routine clinical practice. The figure shows the performance of the model on the composite label against the radiologist’s best assessment of structural abnormality. A total of 15 radiologists (five chest attendings, three chest fellows, and seven general attendings) were asked to read the same set of images. The model outperforms all 15 individual radiologists, attaining a higher sensitivity at the same specificity as each radiologist. The consensus vote across all radiologists is displayed as an additional reader to serve as a single point of comparison to the model. At a fixed specificity of 73%, the model achieves a sensitivity of 71%, while the consensus vote sensitivity is 66%. At the same sensitivity as the consensus vote, the model achieves a positive predictive value of 63%, while the consensus vote positive predictive value is 55%. AUROC, area under the receiver operating characteristic curve; AUPRC, area under the precision–recall curve; DLV, dilated left ventricle; SLVH, severe left ventricular hypertrophy.

We compared the model’s performance on a subset of 408 randomly sampled images (204 from CUIMC and 204 from Stanford) to that of 15 board-certified radiologists. The composite model’s AUROC on this set of images was 0.79. The model outperformed each of the 15 radiologists, achieving better sensitivity at the same specificity attained by a given radiologist. We additionally used the consensus vote label as a proxy for ‘average’ performance. Using the consensus vote as a single point of comparison, we looked at the model’s sensitivity at the same specificity as the consensus vote. The consensus vote across all radiologists had a sensitivity of 66%, a specificity of 73%, and a PPV of 55%. At the same specificity as the consensus vote, the model achieved a sensitivity of 71%. At the same sensitivity as the consensus vote, the model achieved a PPV of 63% (*Figure 4*).

### Saliency mapping

For all 3667 CXRs in the CUIMC test set, we generated LayerCAM visualizations targeting each continuous label at every DenseBlock and TransitionLayer within our DenseNet-121 architecture. These visualizations were systematically reviewed to identify global patterns that emerged consistently across many CXRs.^32^

**Figure 5:**
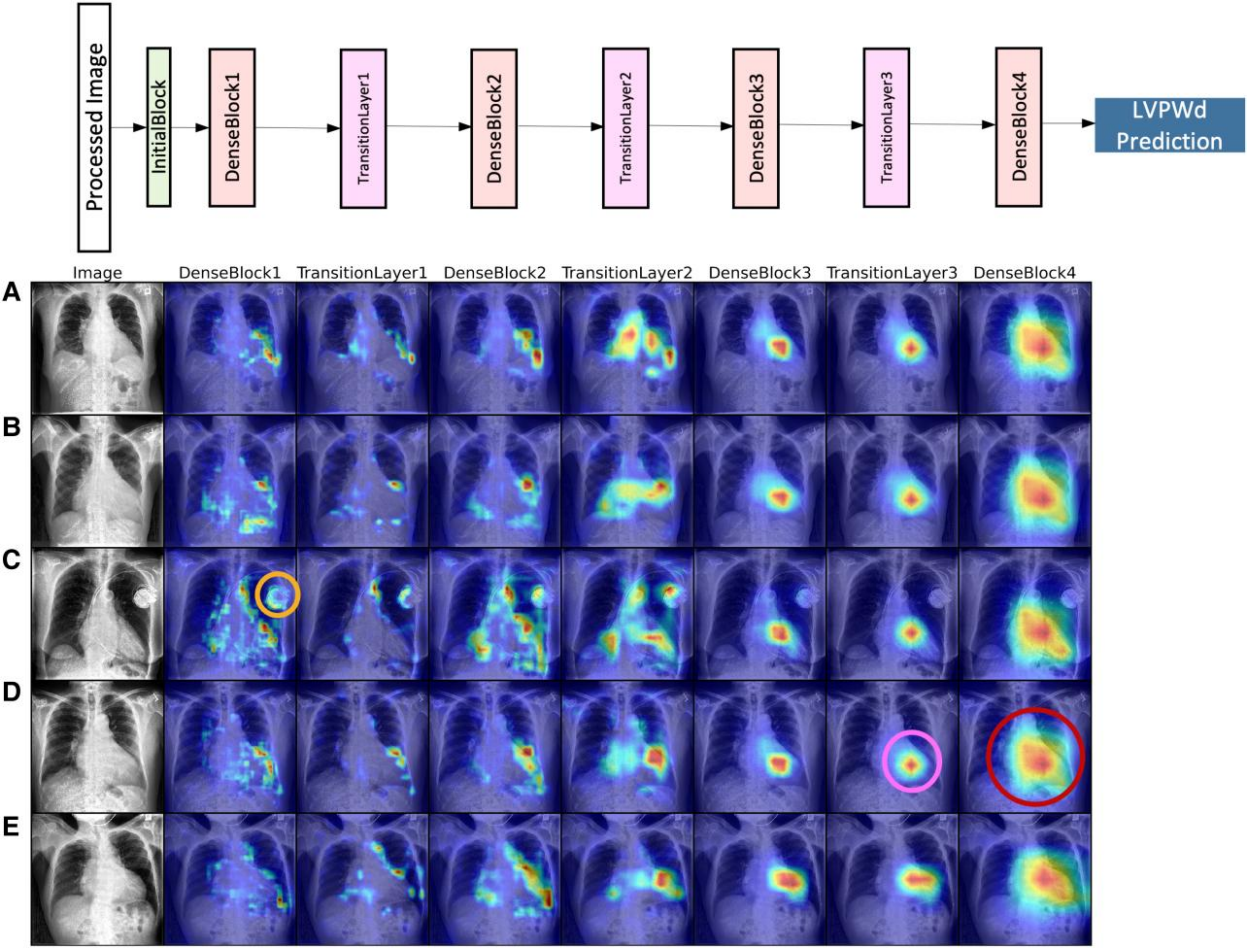
LayerCAM for chest X-rays with respect to left ventricular posterior wall distance at end-diastole continuous label. The figure shows saliency maps for five patients amongst the true positives for the composite label. For each patient, the feature map for each intermediate layer in the model architecture is displayed (the corresponding model layer is indicated above each column). The heatmaps show which areas of the image the model are attending to for making predictions. In analysing all heatmaps of the test set, broad patterns emerged represented by these examples: (orange circle, Patient C) in earlier layers the model can show sensitivity to implanted devices as highlighted in the orange outline for Patient C; (pink circle, Patient D) in intermediate layers, heatmaps are more concentrated within the borders of the cardiac silhouette and often highlighted areas corresponding to the left ventricle as shown in the pink outline; (red circle, Patient D) in the final layer, the model shows sensitivity to a broad area in the centre of the chest X-rays corresponding to the cardiac silhouette as shown in the red outline. LVPWd, left ventricular posterior wall distance at end-diastole.

For any given CXR, the visualizations generated for IVSd, LVIDd, and LVPWd were very similar. *Figure 5* shows LayerCAM heatmaps targeting LVPWd for a sample of five CXRs, each from a distinct patient (Supplementary data online, *Figures S1* and *S2* respectively show the corresponding heatmaps targeting IVSd and LVIDd). As seen in this sample, heatmaps for the deepest layer (DenseBlock4) showed sensitivity to a broad area in the centre of the CXR corresponding to the cardiac silhouette. Meanwhile, at slightly shallower layers (DenseBlock3 and TransitionLayer3), heatmaps were more concentrated within the borders of the cardiac silhouette and often highlighted areas corresponding to the left ventricle. At shallower layers (DenseBlock1 and 2 and TransitionLayer1 and 2), patterns of sensitivity were far less consistent across CXRs, though sensitivity to various regions along the periphery of the cardiac silhouette was regularly observed.

LayerCAM heatmaps often did not show sensitivity to implanted medical devices. However, the model did appear sensitive to PMs particularly at shallower layers (e.g. *Figure 5*, third row; DenseBlock1 and 2 and TransitionLayer1 and 2), though sensitivity to cardiac structures was often more pronounced.

## Discussion

The main findings of this study are as follows: (i) we developed and demonstrated that a deep learning model can accurately identify patients with LV structural abnormalities associated with heart failure using only CXRs (composite SLVH/DLV; AUROC: 0.80); (ii) model performance remains similar when evaluated on data from an external site; (iii) the model outperforms a group of board-certified radiologists for identifying the presence of either structural abnormality, a benchmark never previously achieved; (iv) on CXRs conducted prior to the first echocardiogram, model performance is the same or better across labels, demonstrating promise for using the model as a screening tool; and (v) saliency maps of shallower layers reveal that the model is sensitive to the cardiac silhouette and areas around the left ventricle.

The principal research question we sought to address was whether CXRs contain enough information for the accurate detection of structural abnormalities indicative of structural heart disease. The model we built demonstrates good performance on all three structural abnormality labels we assessed including SLVH (AUROC: 0.79), DLV (AUROC: 0.80), and a composite label representing the union of both labels (AUROC: 0.80).

Assessing model performance using data from independent sites is crucial to ensure that the model is not narrowly applicable for certain populations or learning spurious signal in the training population. The decrease in performance on the SLVH label in the Stanford data set was the most pronounced amongst all labels. Possible causes for performance drops include (i) explicit differences in the input data (e.g. different devices used to conduct CXRs and distinct pre-processing steps prior to saving images), (ii) distinct patient populations (e.g. a particular structural abnormality may be more common at Stanford vs. CUIMC), (iii) differences in practices related to reading echocardiograms, (iv) noisier labels due to more rapid structural changes over shorter periods of time. Machine learning models have been known to be susceptible to these kinds of data distribution shifts.^33–35^ We trained a model to predict the source institution given a data set of CXRs from both institutions and the model had near-perfect accuracy, indicating a detectable difference in data distributions. Another hypothesis is a difference in the patient populations themselves, as can be clearly noted by the much larger prevalences of all labels in the external set. Despite these issues, the model still maintained relatively good accuracy on the composite label in the external set, showing promise that these results could be replicated at other sites as well.

Beyond assessing generalizability, we also sought to assess performance on a population that would better replicate the set of patients who would benefit from heart failure screening. On CXRs conducted in the 12 months prior to a patient’s first echocardiogram, the model had an AUROC of 0.80 on the composite label, establishing that the model maintains performance on a more realistic screening population.

Explainability of deep learning models is critical for not only building trust in model outputs but also verifying that the model is not learning from confounding factors. The saliency maps showed that the model was sensitive to the broad cardiac silhouette and, at shallower layers, structures of the left heart. At shallower layers, the model was also slightly sensitive to the presence of implanted medical devices such as PMs. However, these layers still showed strong sensitivity to areas around the cardiac silhouette, showing that the signal coming from structures of the heart was most important and present in all images. These findings are consistent with results showing that the model maintains performance in the absence of populations with transplants or implantable devices. Practitioners should consider visualizing shallower layers in their models, particularly when interested in identifying sensitivity to fine-grained structures to which deeper layers are less responsive.

After establishing that the model can accurately identify structural abnormalities, it was important to evaluate how well the model performed against the best clinical interpretation of structural abnormalities by experts. The model outperformed all radiologists on the subset of 408 films, using the composite label as the gold standard for structural abnormality. At the same specificity, the model outperformed the consensus vote of all radiologists by 5% on sensitivity. While radiologists are not formally trained to diagnose SLVH and DLV, the study attempted to make the comparison as favourable to radiologists as possible. The CXRs used only included SLVH and DLV pathologies and excluded any other pathologies like dilated right ventricle that may be another cause of cardiomegaly. Radiologists were told the comparison only had to do with cardiomegaly and given no time constraints. Given this prompting, they could solely focus on the characteristics of the cardiac silhouette. During routine clinical interpretation, radiologists may be less attentive to the heart, resulting in potentially worse performance. To date, studies have only shown AI models to be inferior to radiologists in diagnosing cardiac pathology from radiologic modalities. Our study establishes that a model trained on higher quality labels (echocardiographic wall/chamber measurements) can outmatch the best possible clinical evaluation of cardiomegaly using the same image. This is a strong indication that there is a finer signal in CXRs, which can be used to detect more actionable pathologic findings than cardiomegaly.

The European Society of Cardiology (ESC) guidelines^36^ have recently advocated for more studies on screening for heart failure in asymptomatic patients. The American College of Cardiology (ACC), American Heart Association (AHA), and ESC all provide Class I indications for conducting CXRs in suspected new-onset heart failure. The ACC/AHA guidelines^4^ though have noted the limited sensitivity and specificity CXRs have in the diagnosing heart failure. Machine learning models trained on high-quality labels derived from echocardiograms provide a way to significantly improve the diagnostic accuracy of a cheap, prevalent diagnostic test that already holds a Class I indication.

The retrospective analyses presented herein are a necessary first step towards establishing the viability of detecting LV abnormalities from CXRs. This opens avenues for designing an approach to validating such a model prospectively. A prospective deployment strategy in which patients who undergo CXRs may be flagged by the model as high risk for structural abnormalities and referred for echocardiography may be an attractive strategy for diagnosing heart disease earlier, but how radiologists should best interact with such a technology requires further investigation. Since CXRs are a widely deployed clinical test across a variety of clinical settings, they may function as the ideal data modality to capture a certain proportion of this underdiagnosed population.

## Limitations

Firstly, these continuous echocardiographic measurements are subject to interpretation and known to have inter-observer variability. We attempted to mitigate this issue by modelling this noise in the form of a variance parameter. The method used to identify PMs, HTs, and LTs was reliant on using a specific set of keywords based on radiology reports. This method may not have captured all patients in each subpopulation, but clinician review found the method to have high precision after reviewing 150 studies for each keyword set. We were unable to similarly identify patients with sternotomy wires. In terms of the generalizability of our model, we were only able to obtain data from one external site. Machine learning models trained on medical images are known to be susceptible to data set shifts from numerous sources including the usage of different devices for recording a CXR with distinct settings and differing patient populations.^33–35^ To truly assess the robustness of the model, a larger multi-site study would need to be conducted. To more closely match the population present in a hypothetical screening program, we assessed performance on a subset of the training data consisting of CXRs conducted prior to the first echocardiogram. A limitation of this approach is that these patients were within 1 year of echocardiographic diagnosis and may not be representative of patients earlier in the disease course. Finally, while saliency mapping techniques serve as useful methods for auditing and verifying that the model is sensitive to realistic features in the image, deep learning methods remain opaque in terms of explaining individual predictions. This poses a challenge for building trust amongst users.

## Conclusions

To date, studies building machine learning models to evaluate cardiac pathology on CXRs have focused on predicting cardiomegaly, a diagnosis that is poorly predictive of cardiac disease. Our study is the first to demonstrate that CXRs can be used for detecting structural abnormalities associated with Stage B^37^ or worse heart failure, potentially expediting diagnosis and improving clinical outcomes. To our knowledge, it is also the first study showing an AI model can outperform radiologists in the detection of cardiac pathology. These results show early promise that machine learning may enable effective screening using cheap, prevalent imaging to identify patients with undiagnosed heart failure, potentially even in pre-symptomatic Stage B, through earlier detection of structural abnormalities.

## Supplementary Material

ehad782_Supplementary_Data

## Data Availability

All chest X-rays and metadata information corresponding to each chest X-ray (including echocardiogram labels) from CUIMC will be made public for use by the broader research community. Code used to train/evaluate the models and the trained model will be made available on a public GitHub repository. For more information about data access and code, visit this link https://members.dbmi.columbia.edu/CRADLE/LVHnet.html.

## Pre-registered Clinical Trial Number

None supplied.
