# Supplementary material for: Deep learning to detect left ventricular structural abnormalities in chest X-rays
Source: Eur Heart J. 2024 Mar 20;45(22):2002–12. doi: 10.1093/eurheartj/ehad782 (PMC11156488; doi:10.1093/eurheartj/ehad782)
Supplement: ehad782_Supplementary_Data [file ehad782_supplementary_data.zip › SupplementalMethods.docx]

**SUPPLEMENTAL METHODS AND ANALYSES**

**METHODS**

***DATA PREPROCESSING***

As noted in the main text, CXRs were cropped to a 1:1 aspect ratio and downsampled to a 224-by-224 pixel image using bicubic interpolation. Images were downsampled to ensure that images were the same size and to reduce memory/runtime constraints for model training. In additional experiments, it was noted that downsampling the image to a larger image size (e.g. 448-by-448 pixels) did not have a notable effect on performance. We applied contrast-limited adaptive histogram equalization^1^ to each image to improve local contrast of each CXR. All CXRs were first normalized using the overall mean and variance of the training set and then gaussian noise was added to each image using normal distribution with mean 0 and variance 0.05 to improve generalization performance.

***MODEL ARCHITECTURE***

The DenseNet-121^2^ architecture was used as the backbone of the model as it has been shown to learn effective representations of CXRs using a series of convolutions and residual connections^3,4^. Several model architectures were tested as it has been shown in prior work that architecture may affect performance^5^. We tested several versions of the EfficientNet^6^ architecture and it was determined that there was no significant difference in performance across architectures, so we opted to use the simplest of the architectures in DenseNet-121. The representation of the image at the last layer of the neural network along with sex and the continuous age of the patient at the time of the CXR was combined to produce a single data vector. Age was standardized using the training set mean and standard deviation and Sex was encoded using a one-hot encoding. This complete feature vector was then passed to a final feed forward layer. Unlike many existing methods which model binary labels, we focused on modeling continuous echocardiographic measurements. We used the model’s final layer to generate mean and variance parameters for three independent univariate normal distributions, one for each echocardiographic measurement. Using the continuous measurements provides the model with more precise measurements of the underlying structures of the heart which it must identify to effectively detect the presence or absence of each structural abnormality (e.g. the model can learn that a CXR for a patient with an LVPWd of 1.5cm looks distinct from a patient with LVPWd of 2.5cm). Generating a variance allows the model to directly output a parameter for its level of confidence in the predicted value. Subsequently, these distributions are converted to probabilities for each of the three binary labels indicating structural abnormalities (SLVH, DLV, Composite SLVH/DLV). This procedure is demonstrated in **Supplementary Figure 3**.

The model’s final linear layer generates mean and variance parameters for three independent univariate normal distributions, one for each echocardiographic measurement. Using these individual CXR-specific distributions, these distributions are converted to probabilities of each continuous value being above thresholds that define pathology (1.5 cm, 5.9 cm and 1.5 cm respectively for IVSd, LVIDd and LVPWd for men). Using these probabilities, a predicted probability for each individual pathology can be determined using basic probability rules as shown in the figure.

***MODEL TRAINING***

The model was trained using maximum likelihood. The normal distributions parameterized by the model’s output were used to calculate the log-likelihood of each continuous label. The label log-likelihoods were summed and averaged over patients to obtain the total log-likelihood, which was negated to yield a loss function. The loss was calculated over minibatches of CXRs and minimized using stochastic gradient descent (SGD). We saved the model with the largest total log-likelihood on the validation set and finally evaluated model performance on the test set. Along with early stopping we used the following hyperparameters for learning with the ADAM optimizer: [batch size: 32, epochs: 50, learning rate: 1e-3]*.* The normal distributions for each echocardiographic measurement were used to calculate probabilities for each discrete label (SLVH, DLV, Composite SLVH/DLV). See **Supplementary Figure 3** for an example of this procedure.

***GENERATING LAYER-CAMs***

**In a convolutional neural network, each intermediate layer produces a different representation of the original image. For models trained on natural images, it has been shown that in earlier layers, the model may learn to detect lines and edges in the image while later layers are able to detect more complicated structures (e.g. detailed facial features).**

**Class Activation Maps (CAMs) are often used as a method of assessing regions within an image to which a model’s output is most sensitive. CAM visualizations are created by manipulating the activation maps output by a convolutional layer. For models trained on natural images, practitioners commonly use CAM algorithms best suited to visualizing the deepest layer closest to the final output. Activation maps at this depth are small compared to the input image (7x7 pixels in our model); they thus have relatively large receptive fields responsive to large-scale structures such as whole objects. When modeling medical images, CAM visualizations for the deepest layer are often vague (e.g. high sensitivity in entire lung fields when detecting lobar pneumonia^3^). In our case, we are interested in the model’s sensitivity to finer-grained details within CXRs. We, therefore, visualized layers at various depths using LayerCAM^7^, a CAM algorithm developed specifically for this purpose.**

**To visualize a given layer, LayerCAM combines activation maps with gradients backpropagated from a specific element of the model’s final output. This produces a heatmap highlighting the regions of the input image that the present layer relied on most to influence the output value. For each visualization, we backpropagate from a single element of the final output. We choose only those elements corresponding to the mean parameters used to estimate the likelihood of each echocardiographic measurement.**

**Explainability techniques, like CAM, can be misleading when used to interrogate a model on an image-by-image or patient-by-patient basis^8^. To guard against this, we generated visualizations for all CXRs in the CUIMC test set and reviewed them in aggregate to identify patterns of sensitivity which persisted recognizably across the population.**

***EVALUATION METRICS AND BOOTSTRAPPING***

We obtained 95% confidence intervals (CIs) for all statistics by resampling predicted probabilities and true probabilities and using a reverse percentile bootstrap. In the test set, certain patients may have multiple CXRs, causing them to be overrepresented. For all metrics, we randomly resampled a single CXR per patient in the test set 100 times and report the mean and confidence interval when resampling using this method in all relevant Supplementary Tables.

***RADIOLOGIST COMPARISON PROMPT***

The following prompt was given to each radiologist accompanied by the set of full-resolution CXRs.

“We have built a deep learning model on patients who have had CXRs and echocardiograms done within one year of the model. This model learns to detect severe left ventricular hypertrophy and dilated left ventricle from CXR images. In this study we ask you to look at roughly 400 CXRs completed at Columbia and Stanford University. All of these images are PA frontal films done within one year of an echocardiogram. The only evaluation requested from you is if you believe there is an enlarged cardiac silhouette on CXR. Essentially, we are attempting to approximate your clinical read and if you believe there to be cardiomegaly. We acknowledge that since there are no priors, certain processes such as pericardial effusion cannot be ruled out. Please read these as you would any clinical CXR. After we have received the results from all reading physicians, we will compare the accuracy in detecting cardiac structural abnormalities (specifically severe left ventricular hypertrophy and dilated left ventricle) of radiologists versus the deep learning model. We look forward to including you in this study and sharing the results with you!”

**ADDITIONAL ANALYSES**

***CROSS-VALIDATION***

We conducted 20-fold leave-one-out cross validation to assess the variance of our test set performance. Cross validation can provide a more precise estimate of performance since we can evaluate held-out performance over the entire dataset. In this analysis, we used a stratified split across patients where the percentage of the positive cases of the Composite LSVH/DLV label was approximately equal across all folds. Subsequently, we trained 20 different models using the same hyperparameter settings (batch size: 32, epochs: 50, learning rate: 1e-3). We report the mean and the standard deviation across all of the test folds in **Supplementary Figure 9**. The results show that performance is similar to what was reported in our original train/validation/test split. When sampling a single CXR per patient, the variance of performance across folds is reduced further with performance as follows: .77 [95% CI, .73 – .80] for SLVH, .82 [95% CI, 77 - .87] for DLV and .79 [95% CI, .75 - .82] for the Composite SLVH/DLV label.

***CALIBRATION OF CONTINUOUS ECHOCARDIOGRAPHIC MEASURES***

To assess the model’s ability to accurately predict the echocardiographic measurements from a CXR, we plotted the predicted values using the model’s predicted mean for each measurement against the true values. **Supplementary Figure 10** shows three different plots (one for each continuous label), showing the predicted mean value of each continuous measure against its true value in the test set. In each plot, a lowess regression is also estimated, indicated by the red line. These figures show that the correlations are relatively weak with r-squared scores of 0.24, 0.23, and 0.13 for IVSd, LVPWd and LVIDd respectively. It is worth noting that echocardiography measurements can be noisy with interobserver variability so strong correlation may be difficult to achieve given what we consider gold standard data still may have some degree of noise. The root mean squared error (RMSE) values for each of these measures are respectively 0.20, 0.18 and 0.56. The mean absolute error (MAE) values are even smaller; they are 0.15, 0.14 and 0.43, respectively. This indicates that on average, the model can predict within .15 and .14 cm of the true values for IVSd and LVPWd. The RMSE/MAE values coupled with the binary prediction results indicate that the model is still valuable for detecting cardiac pathology.

***CALIBRATION OF BINARY LABELS***

We additionally evaluated the calibration of the binary predictions for SLVH, DLV and the Composite label. **Supplementary Figure 11** shows a calibration curve and the accompanying distribution of the predicted probabilities for each of the three labels. In plotting calibration curves, we apply quantile binning such that each point on the curve corresponds to approximately the same number of data points. Since most of the predicted probabilities are between .0 and .2, there are more points in this region of the curve. Without any additional post-hoc calibration, the model is under-calibrated with predicted probabilities underestimating the true risk. Since the model was trained on the echocardiogram continuous measurements rather than directly on the binary labels, it is difficult for the model to be naturally well-calibrated. While the continuous measures provide more signal to the model, they may create such challenges when predicted probabilities for downstream binary labels are derived from continuous predictions as shown in **Supplementary Figure 3**. However, there are numerous methods to correct for such discrepancies in calibration. We applied a standard post-hoc calibration technique known as isotonic regression and show in **Supplementary Figure 12** that the calibration of predictions for all three labels is much improved. After applying this method, the model provides predictions on the test set that are much more consistent with the true underlying risk.

***STATISTICAL TESTING FOR MODEL VERSUS RADIOLOGIST PERFORMANCE***

To assess whether there was a significant difference between model performance and the consensus radiologist assessment, we applied McNemar’s test^9,10^ as has been done in prior work^11^. The objective of this test is to assess whether there is a difference in the error rates of two diagnostic tests when evaluated on the same data.

We may use McNemar’s test to compare the diagnostic performance of the model and consensus vote of radiologists. The main challenge with applying this test is that we must specify some threshold to convert the model’s risk scores to binary predictions. McNemar’s test is often applied in cases where this threshold can be explicitly shared between two classification models. In our case, we may specify a threshold by enforcing that the consensus label’s sensitivity, specificity or any other metric of interest matches that of the model.

We determine an operating point for the model based on the consensus vote sensitivity. At the chosen operating point, the sensitivity of the model is 66%, the same as that of the consensus vote of the radiologists. The null hypothesis is that the error rates of the diagnostic tests do not differ. When applying this test, we get the following contingency table:

|  | *Consensus Vote* | |
| --- | --- | --- |
| *Model* | ***A:*** *number misclassified by neither*  *256* | ***B:*** *number misclassified by consensus but not by model*  *55* |
|  | ***C:*** *number misclassified by model but not by consensus*  *33* | ***D:*** *number misclassified by both*  *64* |

The test statistic is $\frac{\left( \left| B-C \right|-1 \right)^{2}}{B+C}=5.01$. The test statistic is known to be distributed as a Chi-square distribution with 1 degree of freedom. Given this, the p-value for this test is 0.025 and we can reject the null hypothesis at a significance level of 0.05.

We caution that any choice of statistical test has drawbacks in this scenario. For example, the consensus vote across radiologists can be estimated from the existing data accurately, but it is not always an accurate reflection of true clinical practice. Nonetheless, there is often an adjudication process which may involve multiple radiologists in cases of uncertain diagnoses. Additionally, this test does not fully account for the sample size across all the independent radiologist readings since the consensus vote is a summary across all the radiologists.

Other methods for assessing the statistical difference include summarizing the radiologist performance as a summary ROC curve^12^. The main challenge with this method is that it does not yield a full AUROC curve and requires extrapolation to regions outside of the radiologists’ observed true positive and false positive rates. In our case, this yields a curve defined in a very restricted region of the domain since our observed radiologist false positive rates span a small portion of the domain.

**SUPPLEMENTARY FIGURE AND TABLE LEGENDS**

Supplementary Figure 1 LayerCAM for CXRs with respect to IVSd continuous label. The figure shows saliency maps for the IVSd continuous label. Similar patterns emerge as in Figure 5, with the model attending to more finer regions of the left heart in shallower layers.

Supplementary Figure 2 LayerCAMs for CXRs with respect to LVIDd continuous label. The figure shows saliency maps for the LVIDd continuous label. In contrast to the saliency maps for IVSd and LVPWd, the feature maps in the intermediate layers cover broader areas of the left side of the heart. Since the left ventricular internal diameter at end-diastole is a measurement of the full diameter of the left ventricular region of the heart, this is consistent with what the model is aiming to predict.

Supplementary Figure 3 Model Prediction: Conversion from Continuous to Binary Prediction. The model’s final linear layer generates mean and variance parameters for three independent univariate normal distributions, one for each echocardiographic measurement. Using these individual CXR-specific distributions, these distributions are converted to probabilities of each continuous value being above thresholds that define pathology (1.5 cm, 5.9 cm, and 1.5 cm respectively for IVSd, LVIDd and LVPWd for men). Using these probabilities, a predicted probability for each individual pathology can be determined using basic probability rules as shown in the figure.

Supplementary Figure 4 Model Performance on CUIMC Test Set Across Demographic Subpopulations: Ethnicity, Race, Age, Sex. The figure shows model performance in demographic subpopulations along with 95% bootstrapped confidence intervals. There is no significant difference in model performance on the Composite label across non-Hispanics and Hispanics, nor across Black and White patients. Performance was also similar across males and females, as well as across different age groups. This finding suggests that the model may perform well even within populations in which patients are at different baseline levels of risk.

Supplementary Figure 5 Distribution of Months Between CXR and Echocardiogram for each Continuous Echocardiogram Measurement. For each continuous measurement, at least 70% of chest X-rays are paired with echocardiograms conducted within 6 months of the chest X-ray indicating that all three measurements are typically derived from echocardiograms in close temporal proximity to the chest X-ray.

Supplementary Figure 6 Distribution of Months Between CXR and Echocardiogram for different subsets of data. For each CXR, we can find the furthest echocardiogram temporally from which a measurement was derived. These plots represent the absolute difference in months between each CXR and echocardiogram pair in three different subsets of data: 1) the train and test set 2) the dataset of CXRs prior to first echocardiogram 3) the Columbia CXRs read by radiologists. The trend is similar to Supplementary Figure 5, where most of the CXRs and echocardiogram pairs are conducted in close temporal proximity to each other (within 6 months).

Supplementary Figure 7 Changes in Echocardiogram Continuous Measurements between Successive Echocardiograms. There is no significant difference in the distributions of continuous measurements between echocardiograms taken 0-1 month apart, 0-3 months apart, 3-6 months apart, 6-9 months or 9-12 months apart. This demonstrates that, at least in our dataset, these measurements do not change more or less drastically in shorter periods of time compared to longer periods of time within the 1 year window.

Supplementary Figure 8 Performance on Test Set Stratified by Months between CXR and Echo. The figure shows model performance stratified by months between CXR and Echo along with 95% bootstrapped confidence intervals. There is no significant difference in performance when stratifying by months between CXR and echocardiogram. For the Composite label, the performance is very similar across different strata, except for 6-9 months, but the confidence intervals show is likely random chance.

Supplementary Figure 9 Performance on 20-fold Cross Validation. In an analysis of the same dataset split using 20-fold cross validation across patients, the model performance is similar to that of the original test set with the confidence interval shrinking further when analyzed on a per-patient basis in each test fold. The model performance is .77 [95% CI, .73 – .80] for SLVH, .82 [95% CI, 77 - .87] for DLV and .79 [95% CI, .75 - .82] for the Composite SLVH/DLV label when sampling a single CXR per patient per test fold indicating that the model maintains strong performance even when assessing test statistics using cross validation. Supplementary Figure 10. Predicted vs Observed Plots for each Continuous Echocardiogram Measurement. To assess the model’s ability to predict each continuous echocardiogram label, we plotted the predicted versus observed values for each of the three measures. A loess regression of the scatterplot is also estimated in red. The model shows relatively weak correlation with r-squared values ranging from 0.13 to 0.24. However, the RMSE values are 0.24, 0.23 and 0.56 for IVSd, LVPWd and LVIDd respectively. The mean absolute error values are 0.15, 0.14 and 0.43, respectively. These results coupled with the binary prediction results, indicate that the model is still useful for detecting cardiac pathology among a set of CXRs.

Supplementary Figure 11 Calibration Curves for Binary Label Prediction. Without any additional post-hoc calibration, the model is under-calibrated with predicted probabilities underestimating the true risk. Since the model was trained on the echocardiogram continuous measurements rather than directly on the binary labels, it is difficult for the model to be naturally well-calibrated. When plotting the calibration curves, we apply quantile binning so each point on the calibration curve represents approximately the same number of data points.

Supplementary Figure 12 Calibration Curves for Binary Label Prediction after Isotonic Regression. After applying isotonic regression, a post-hoc calibration approach, the model calibration improves significantly. The mean predicted probability is much closer to the true risk.

**Supplementary Table 1 Regular expression used to identify subpopulations.** To identify key subpopulations, we used a set of regular expressions for each population. If there were any mentions of these expressions in the radiology report corresponding to a CXR, the CXR was determined to be part of said subpopulation.

**Supplementary Table 2 Performance of Logistic Regression and Gradient-Boosted Trees fit using Age and Sex for each binary outcome.** For determining that the model was, indeed, using signal from the CXRs, we fit simple logistic and tree-based models using just age and sex as input features predicting each of the three binary outcomes: SLVH, DLV and the Composite label. **Age and sex alone were poor predictors for all three of the labels with an AUROC ranging from 0.51 to 0.59.**

**Supplementary Table 3 Model Performance on CUIMC Test and Stanford External Dataset.** The table shows model performance on the CUIMC test set and Stanford external set on (1) all CXRs in the test set and (2) one sampled CXR per test set. Since one patient may have multiple CXRs, we computed the performance after sampling a single CXR per test set as well. The performance is the same or better in all cases after sampling a single CXR per patient.

**Supplementary Table 4 Model Performance on CXRs before first echocardiogram. On the population of patients with CXRs prior to first echocardiogram, the model maintained an AUROC of 0.80 [95% CI, 0.75-0.86] on the Composite label. The performance for DLV is much higher (0.97) likely due to sampling variance as the prevalence of DLV in this population is very low (0.7%).**

**Supplementary Table 5 Model Performance on Subpopulations.** When looking at performance within each subpopulation, there was no significant difference in the Composite label performance compared to the overall population performance.

**Supplementary Table 6 Subpopulation Characteristics.** Data reported as mean ± SD or n (%). The only patient-level static characteristic is Sex. All other statistics are summarized on a per CXR-echocardiogram pair basis (including age as it may change across different CXRs for the same patient).

**Supplementary Table 7 Distribution of Race, Ethnicity, Age and Sex in CUIMC Test Set.** We extracted the best available race and ethnicity information from our patient records for the test set. A significant complication of collecting this data at CUIMC is that patients can opt out of self-identification of race/ethnicity. While this is a patient-centered approach, it results in a significant portion of patients being labeled “unknown” or “declined”. The only patient-level non-static characteristic is Age. All other statistics are counted on a per-patient basis.

**Supplementary Table 8 Model Performance on Subpopulations: Sensitivity, Specificity, NPV and PPV using Youden’s Threshold.** We computed all four statistics using Youden’s threshold for each of the subpopulations to provide additional context for how the model performs at an individual point on the AU-ROC curve.

**REFERENCES**

1. Pizer SM, Amburn EP, Austin JD, Cromartie R, Geselowitz A, Greer T*, et al.* Adaptive histogram equalization and its variations. *Computer vision, graphics, and image processing* 1987;**39**:355-368. doi:

2. Huang G, Liu Z, Van Der Maaten L, Weinberger KQ. Densely connected convolutional networks. In: *Proceedings of the IEEE conference on computer vision and pattern recognition*. *2017*, p.4700-4708.

3. Rajpurkar P, Irvin J, Zhu K, Yang B, Mehta H, Duan T*, et al.* Chexnet: Radiologist-level pneumonia detection on chest x-rays with deep learning. *arXiv preprint arXiv:1711.05225* 2017. doi:

4. Zhou L, Yin X, Zhang T, Feng Y, Zhao Y, Jin M*, et al.* Detection and Semiquantitative Analysis of Cardiomegaly, Pneumothorax, and Pleural Effusion on Chest Radiographs. *Radiol Artif Intell* 2021;**3**:e200172. doi: 10.1148/ryai.2021200172

5. El Omary S, Lahrache S, El Ouazzani R. Detecting heart failure from chest X-ray images using deep learning algorithms. In: *2021 3rd IEEE Middle East and North Africa COMMunications Conference (MENACOMM)*. *2021*, p.13-18. IEEE.

6. Tan M, Le Q. Efficientnet: Rethinking model scaling for convolutional neural networks. In: *International conference on machine learning*. *2019*, p.6105-6114. PMLR.

7. Jiang P-T, Zhang C-B, Hou Q, Cheng M-M, Wei Y. Layercam: Exploring hierarchical class activation maps for localization. *IEEE Transactions on Image Processing* 2021;**30**:5875-5888. doi:

8. Ghassemi M, Oakden-Rayner L, Beam AL. The false hope of current approaches to explainable artificial intelligence in health care. *The Lancet Digital Health* 2021;**3**:e745-e750. doi:

9. Hawass N. Comparing the sensitivities and specificities of two diagnostic procedures performed on the same group of patients. *The British journal of radiology* 1997;**70**:360-366. doi:

10. Dietterich TG. Approximate statistical tests for comparing supervised classification learning algorithms. *Neural computation* 1998;**10**:1895-1923. doi:

11. Brinker TJ, Hekler A, Enk AH, Berking C, Haferkamp S, Hauschild A*, et al.* Deep neural networks are superior to dermatologists in melanoma image classification. *European Journal of Cancer* 2019;**119**:11-17. doi:

12. Oakden-Rayner L, Palmer L. Docs are ROCs: a simple off-the-shelf approach for estimating average human performance in diagnostic studies. *arXiv preprint arXiv:2009.11060* 2020. doi:
