# Supplementary material for: Deep learning to detect left ventricular structural abnormalities in chest X-rays
Source: Eur Heart J. 2024 Mar 20;45(22):2002–12. doi: 10.1093/eurheartj/ehad782 (PMC11156488; doi:10.1093/eurheartj/ehad782)
Supplement: ehad782_Supplementary_Data [file ehad782_supplementary_data.zip › SupplementaryFigure3.pdf]

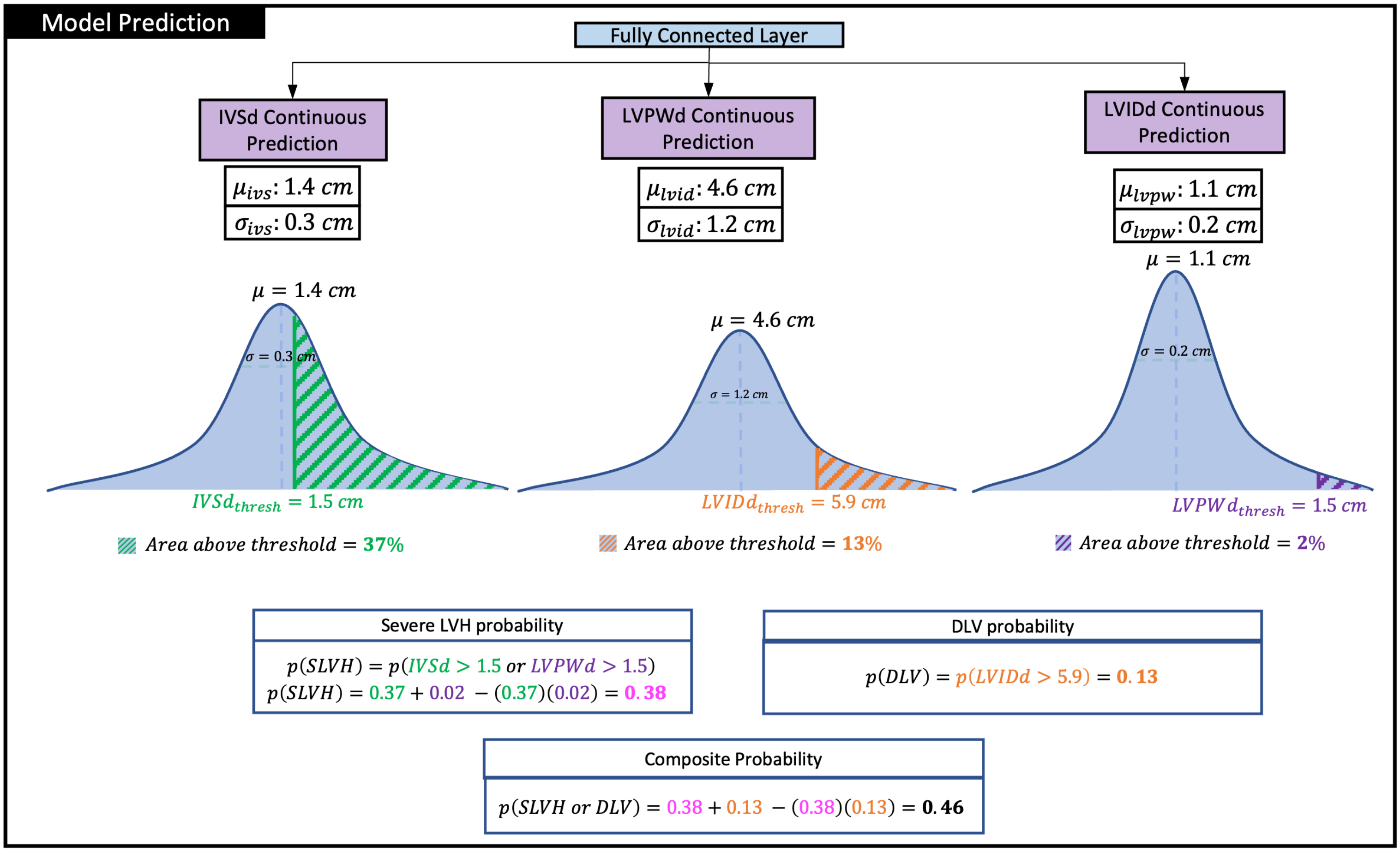
Model Prediction
Fully Connected Layer
IVSd Continuous Prediction
μ_{ivs}: 1.4 cm
σ_{ivs}: 0.3 cm
LVPWd Continuous Prediction
μ_{lvid}: 4.6 cm
σ_{lvid}: 1.2 cm
LVIDd Continuous Prediction
μ_{lvpw}: 1.1 cm
σ_{lvpw}: 0.2 cm
$\mu = 1.4\ cm$
$\sigma = 0.3\ cm$
$IVSd_{thresh} = 1.5\ cm$
Area above threshold = 37%
$\mu = 4.6\ cm$
$\sigma = 1.2\ cm$
$LVIDd_{thresh} = 5.9\ cm$
Area above threshold = 13%
$\mu = 1.1\ cm$
$\sigma = 0.2\ cm$
$LVPWd_{thresh} = 1.5\ cm$
Area above threshold = 2%
Severe LVH probability
$p(SLVH) = p(IVSd > 1.5\ or\ LVPWd > 1.5)$
$p(SLVH) = 0.37 + 0.02 - (0.37)(0.02) = \mathbf{0.38}$
DLV probability
$p(DLV) = p(LVIDd > 5.9) = \mathbf{0.13}$
Composite Probability
$p(SLVH\ or\ DLV) = 0.38 + 0.13 - (0.38)(0.13) = \mathbf{0.46}$
