# Supplementary material for: Deep learning to detect left ventricular structural abnormalities in chest X-rays
Source: Eur Heart J. 2024 Mar 20;45(22):2002–12. doi: 10.1093/eurheartj/ehad782 (PMC11156488; doi:10.1093/eurheartj/ehad782)
Supplement: ehad782_Supplementary_Data [file ehad782_supplementary_data.zip › SupplementaryFigure4Revised.pdf]

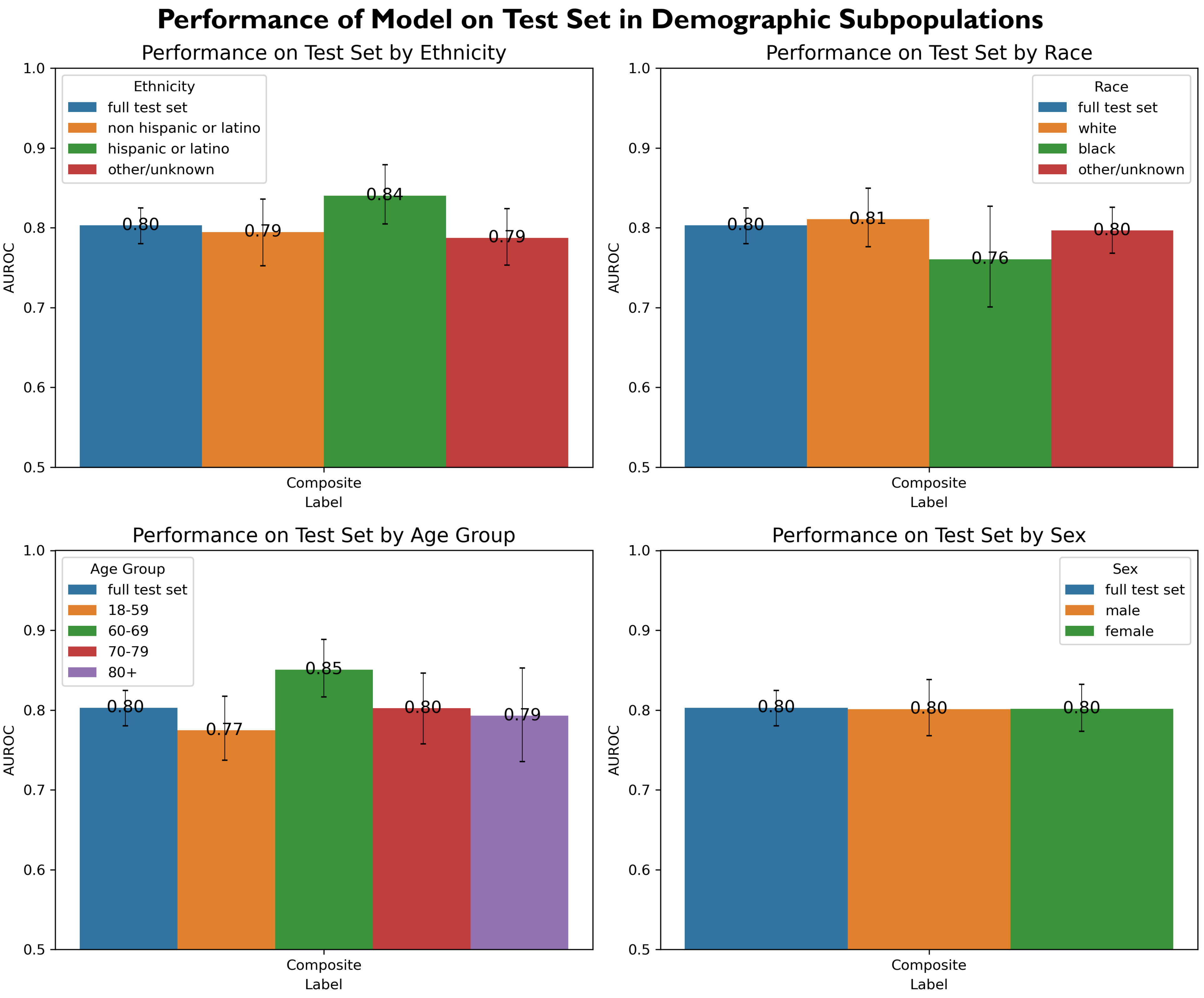

Performance of Model on Test Set in Demographic Subpopulations
Performance on Test Set by Ethnicity
Ethnicity
full test set
non hispanic or latino
hispanic or latino
other/unknown
0.80
0.79
0.84
0.79
AUROC
Composite
Label
Performance on Test Set by Race
Race
full test set
white
black
other/unknown
0.80
0.81
0.76
0.80
Performance on Test Set by Age Group
Age Group
full test set
18-59
60-69
70-79
80+
0.80
0.77
0.85
0.80
0.79
Performance on Test Set by Sex
Sex
full test set
male
female
0.80
0.80
0.80
