# Supplementary material for: Deep learning to detect left ventricular structural abnormalities in chest X-rays
Source: Eur Heart J. 2024 Mar 20;45(22):2002–12. doi: 10.1093/eurheartj/ehad782 (PMC11156488; doi:10.1093/eurheartj/ehad782)
Supplement: ehad782_Supplementary_Data [file ehad782_supplementary_data.zip › SupplementaryFigure5.pdf]

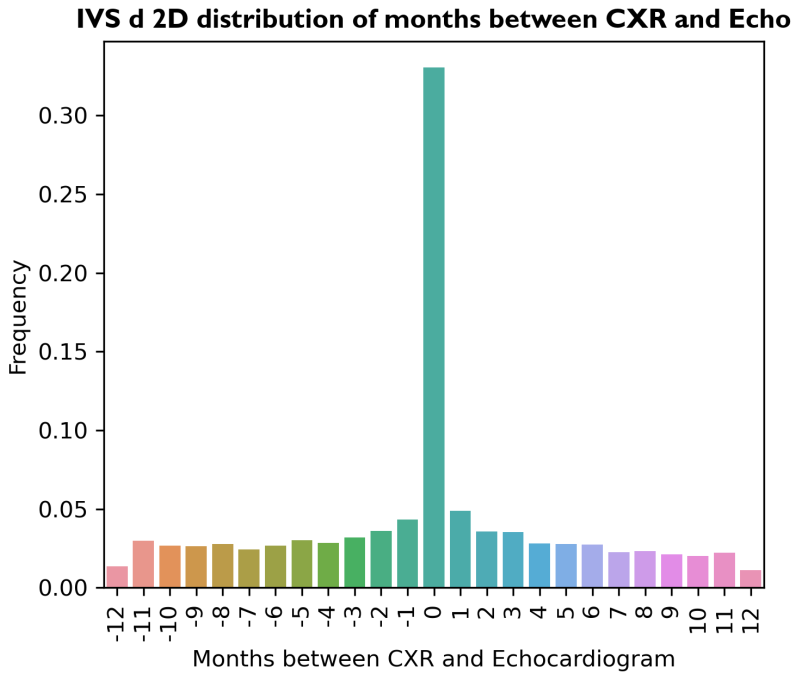
IVS d 2D distribution of months between CXR and Echo
Frequency
0.00
0.05
0.10
0.15
0.20
0.25
0.30
-12 -11 -10 -9 -8 -7 -6 -5 -4 -3 -2 -1 0 1 2 3 4 5 6 7 8 9 10 11 12
Months between CXR and Echocardiogram

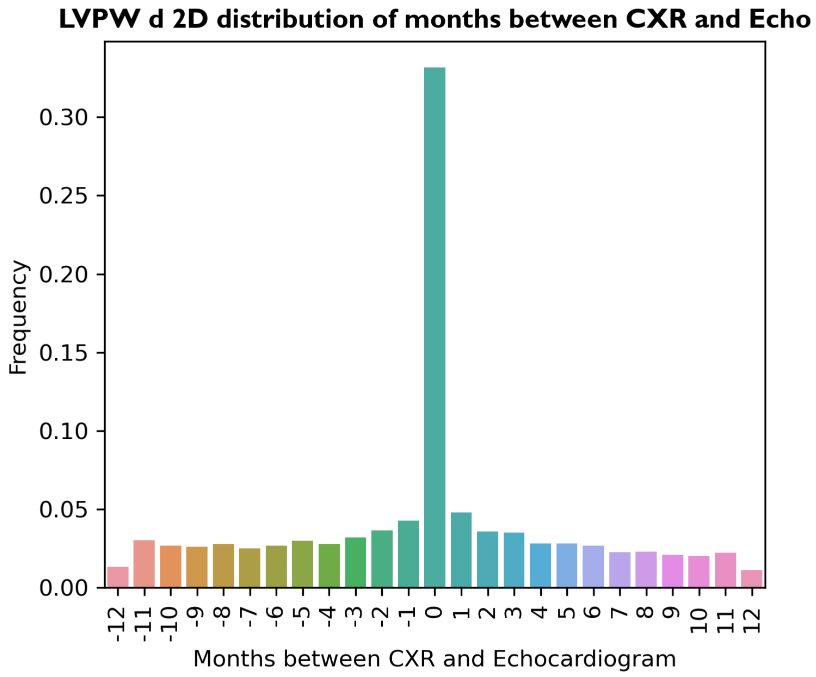
LVPW d 2D distribution of months between CXR and Echo
Frequency
0.00
0.05
0.10
0.15
0.20
0.25
0.30
-12 -11 -10 -9 -8 -7 -6 -5 -4 -3 -2 -1 0 1 2 3 4 5 6 7 8 9 10 11 12
Months between CXR and Echocardiogram

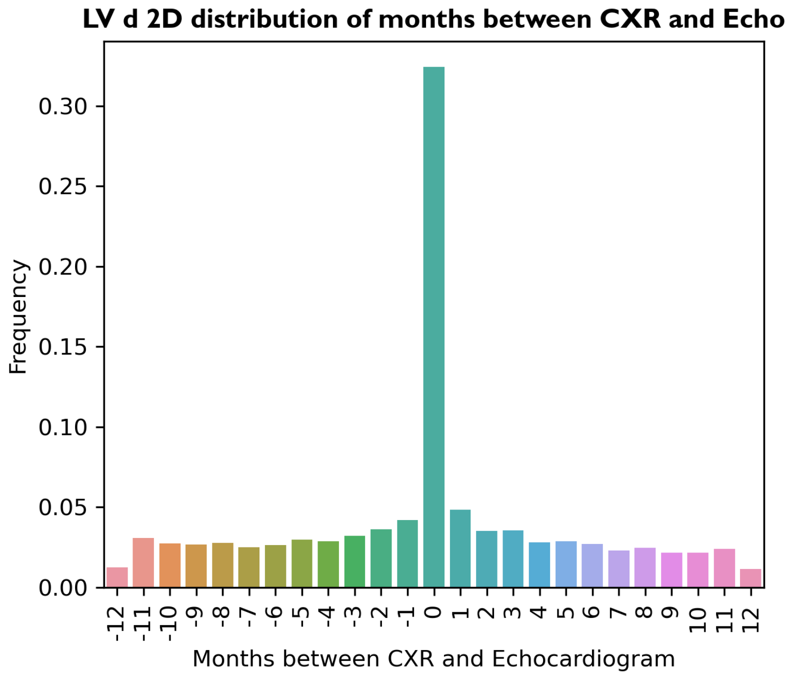
LV d 2D distribution of months between CXR and Echo
Frequency
0.00
0.05
0.10
0.15
0.20
0.25
0.30
-12 -11 -10 -9 -8 -7 -6 -5 -4 -3 -2 -1 0 1 2 3 4 5 6 7 8 9 10 11 12
Months between CXR and Echocardiogram
