# Supplementary material for: Deep learning to detect left ventricular structural abnormalities in chest X-rays
Source: Eur Heart J. 2024 Mar 20;45(22):2002–12. doi: 10.1093/eurheartj/ehad782 (PMC11156488; doi:10.1093/eurheartj/ehad782)
Supplement: ehad782_Supplementary_Data [file ehad782_supplementary_data.zip › SupplementaryFigure6.pdf]

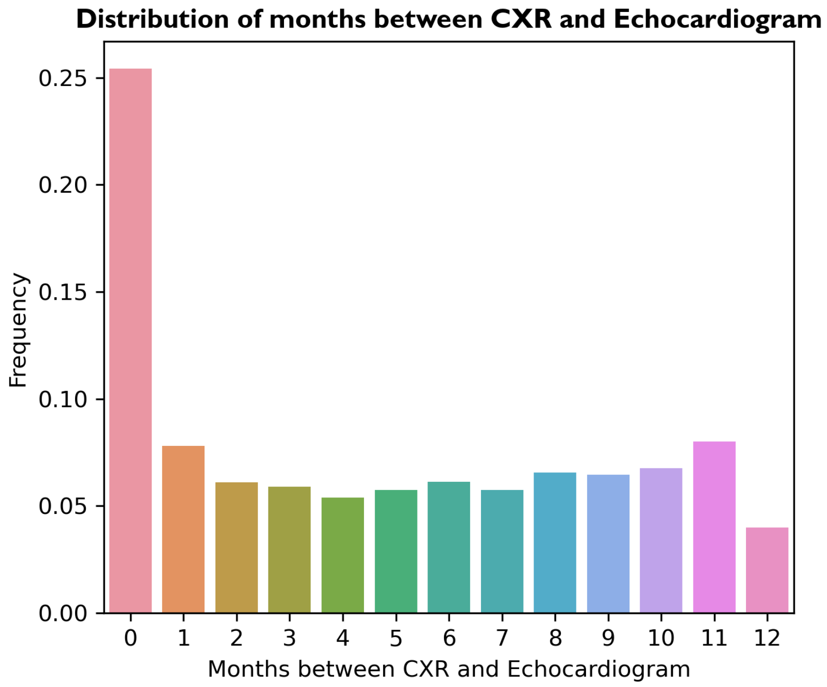
Distribution of months between CXR and Echocardiogram
Frequency
0.00
0.05
0.10
0.15
0.20
0.25
0 1 2 3 4 5 6 7 8 9 10 11 12
Months between CXR and Echocardiogram

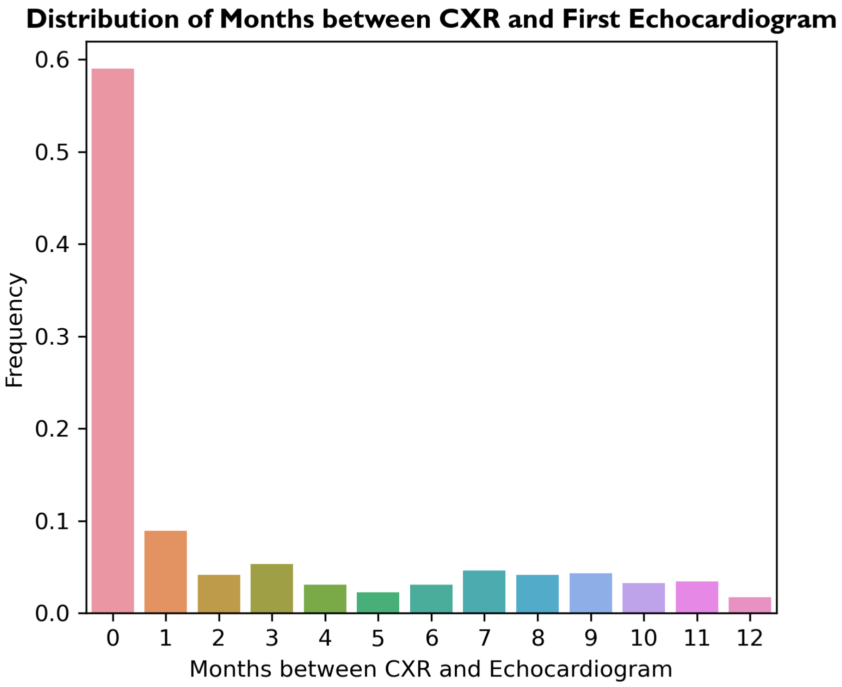
Distribution of Months between CXR and First Echocardiogram
Frequency
0.0
0.1
0.2
0.3
0.4
0.5
0.6
0 1 2 3 4 5 6 7 8 9 10 11 12
Months between CXR and Echocardiogram

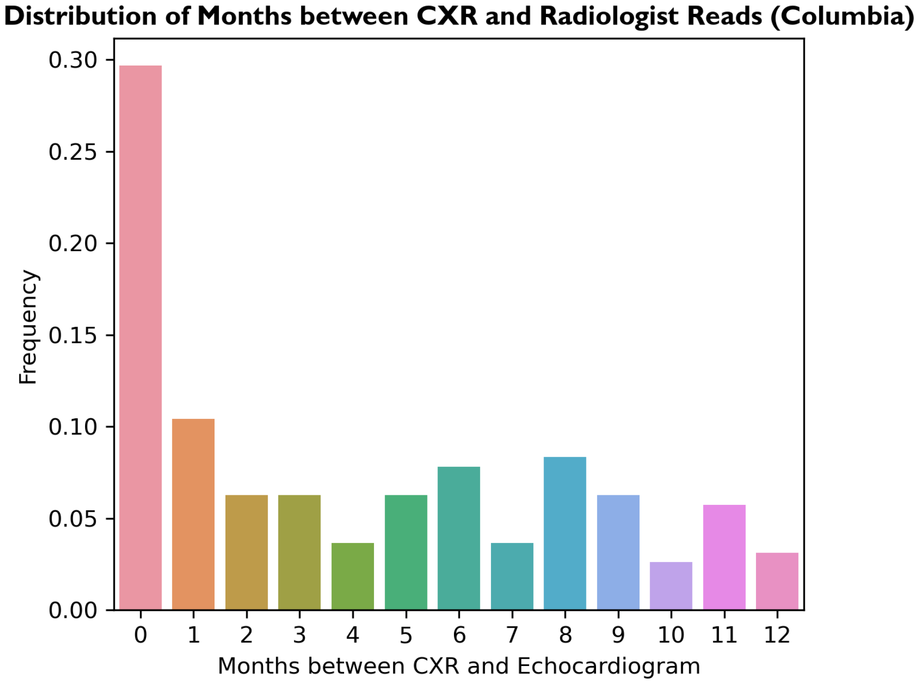
Distribution of Months between CXR and Radiologist Reads (Columbia)
Frequency
0.00
0.05
0.10
0.15
0.20
0.25
0.30
0 1 2 3 4 5 6 7 8 9 10 11 12
Months between CXR and Echocardiogram
