# Supplementary material for: Deep learning to detect left ventricular structural abnormalities in chest X-rays
Source: Eur Heart J. 2024 Mar 20;45(22):2002–12. doi: 10.1093/eurheartj/ehad782 (PMC11156488; doi:10.1093/eurheartj/ehad782)
Supplement: ehad782_Supplementary_Data [file ehad782_supplementary_data.zip › SupplementaryFigure7.pdf]

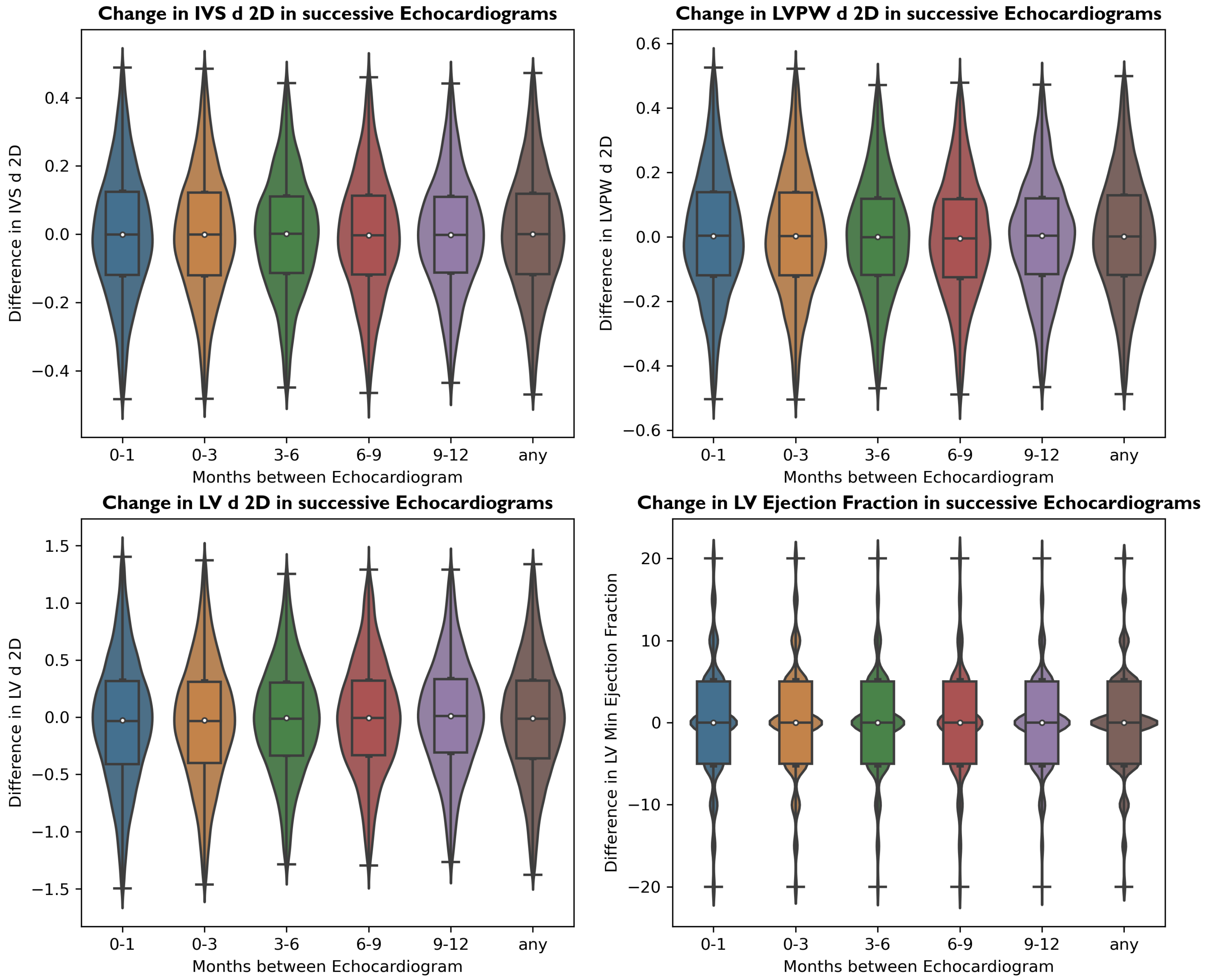

Change in IVS d 2D in successive Echocardiograms
Difference in IVS d 2D
0.4
0.2
0.0
−0.2
−0.4
0-1
0-3
3-6
6-9
9-12
any
Months between Echocardiogram
Change in LVPW d 2D in successive Echocardiograms
Difference in LVPW d 2D
0.6
0.4
0.2
0.0
−0.2
−0.4
−0.6
0-1
0-3
3-6
6-9
9-12
any
Months between Echocardiogram
Change in LV d 2D in successive Echocardiograms
Difference in LV d 2D
1.5
1.0
0.5
0.0
−0.5
−1.0
−1.5
0-1
0-3
3-6
6-9
9-12
any
Months between Echocardiogram
Change in LV Ejection Fraction in successive Echocardiograms
Difference in LV Min Ejection Fraction
20
10
0
−10
−20
0-1
0-3
3-6
6-9
9-12
any
Months between Echocardiogram
