# Supplementary material for: Deep learning to detect left ventricular structural abnormalities in chest X-rays
Source: Eur Heart J. 2024 Mar 20;45(22):2002–12. doi: 10.1093/eurheartj/ehad782 (PMC11156488; doi:10.1093/eurheartj/ehad782)
Supplement: ehad782_Supplementary_Data [file ehad782_supplementary_data.zip › SupplementaryTable1.docx]

| Subpopulation | Patterns |
| --- | --- |
| Pacemakers and Implantable Devices | ^pace, pacemaker, ^device, defibrillator, ICD, PPM, ppm, icd |
| Heart Transplant | HEART REPLACED BY TRANSPLANT, heart transplant, HEART TRANSPLANT, heart tx, heart txp |
| Lung Transplant | LUNG REPLACED BY TRANSPLANT, lung transplant, LUNG TRANSPLANT, lung tx, lung txp |

**Supplementary Table 1 Regular expression used to identify subpopulations**. To identify key subpopulations, we used a set of regular expressions for each population. If there were any mentions of these expressions in the radiology report corresponding to a CXR, the CXR was determined to be part of said subpopulation.
