# Supplementary material for: Deep learning to detect left ventricular structural abnormalities in chest X-rays
Source: Eur Heart J. 2024 Mar 20;45(22):2002–12. doi: 10.1093/eurheartj/ehad782 (PMC11156488; doi:10.1093/eurheartj/ehad782)
Supplement: ehad782_Supplementary_Data [file ehad782_supplementary_data.zip › SupplementaryTable2.docx]

|  |  | **SLVH** | | **DLV** | | **Composite SLVH/DLV** | |
| --- | --- | --- | --- | --- | --- | --- | --- |
|  |  | **AUROC** | **AUPRC** | **AUROC** | **AUPRC** | **AUROC** | **AUPRC** |
| **Model** | **Logistic** | 0.59 | 0.11 | 0.51 | 0.04 | 0.57 | 0.14 |
|  | **GBT** | 0.56 | 0.10 | 0.52 | 0.04 | 0.53 | 0.12 |

Supplementary Table 2 Performance of Logistic Regression and Gradient-Boosted Trees fit using Age and Sex for each binary outcome. For determining that the model was, indeed, using signal from the CXRs, we fit simple logistic and tree-based models using just age and sex as input features predicting each of the three binary outcomes: SLVH, DLV and the Composite label. Age and sex alone were poor predictors for all three of the labels with an AUROC ranging from 0.51 to 0.59.
