# Supplementary material for: Deep learning to detect left ventricular structural abnormalities in chest X-rays
Source: Eur Heart J. 2024 Mar 20;45(22):2002–12. doi: 10.1093/eurheartj/ehad782 (PMC11156488; doi:10.1093/eurheartj/ehad782)
Supplement: ehad782_Supplementary_Data [file ehad782_supplementary_data.zip › SupplementaryTable3.docx]

|  |  | **SLVH** | | **DLV** | | **Composite SLVH/DLV** | |
| --- | --- | --- | --- | --- | --- | --- | --- |
|  |  | **AUROC** | **AUPRC** | **AUROC** | **AUPRC** | **AUROC** | **AUPRC** |
| **CUIMC Test (n=3,667)** | **All CXRs** | .79 [.76, .81] | .19 [.15, .22] | .80 [.77, .84] | .20 [.13, .25] | .80 [.78, .83] | .32 [.27, .36] |
|  | **One CXR per patient** | .80 [.78, .81] | .22 [.19, .25] | .85 [.82, .88] | .31 [.25, .37] | .81 [.8, .82] | .37 [.34, .41] |
| **Stanford External (n=8,003)** | **All CXRs** | .67 [.65, .69] | .17 [.15, .19] | .78 [.76, .79] | .47 [.44, .49] | .76 [.75, .77] | .53 [.51, .56] |
|  | **One CXR per patient** | .68 [.67, .68] | .18 [.17, .19] | .78 [.77, .79] | .45 [.44, .46] | .77 [.76, .77] | .54 [.52, .55] |

Supplementary Table 3 Model Performance on CUIMC Test and Stanford External Dataset. The table shows model performance on the CUIMC test set and Stanford external set on (1) all CXRs in the test set and (2) one sampled CXR per test set. Since one patient may have multiple CXRs, we computed the performance after sampling a single CXR per test set as well. The performance is the same or better in all cases after sampling a single CXR per patient.
