# Supplementary material for: Deep learning to detect left ventricular structural abnormalities in chest X-rays
Source: Eur Heart J. 2024 Mar 20;45(22):2002–12. doi: 10.1093/eurheartj/ehad782 (PMC11156488; doi:10.1093/eurheartj/ehad782)
Supplement: ehad782_Supplementary_Data [file ehad782_supplementary_data.zip › SupplementaryTable4.docx]

|  |  | **SLVH** | | **DLV** | | **Composite SLVH/DLV** | |
| --- | --- | --- | --- | --- | --- | --- | --- |
|  |  | **AUROC** | **AUPRC** | **AUROC** | **AUPRC** | **AUROC** | **AUPRC** |
| **CXR before First Echo**  **(n=583)** | **All CXRs** | .80 [.74, .85] | .16 [.05, .22] | .97 [.94, 1.0] | .34 [.01, .67] | .80 [.75, .86] | .24 [.10, .33] |
|  | **One CXR per patient** | .80 [.78, .81] | .15 [.14, .16] | .97 [.96, .98] | .36 [.35, .37] | .81 [.80, .82] | .25 [.23, .27] |

Supplementary Table 4 Model Performance on CXRs before first echocardiogram. On the population of patients with CXRs prior to first echocardiogram, the model maintained an AUROC of 0.80 [95% CI, 0.75-.0.86] on the Composite label. The performance for DLV is much higher (0.97) likely due to sampling variance as the prevalence of DLV in this population is very low (0.7%).
