# Supplementary material for: Deep learning to detect left ventricular structural abnormalities in chest X-rays
Source: Eur Heart J. 2024 Mar 20;45(22):2002–12. doi: 10.1093/eurheartj/ehad782 (PMC11156488; doi:10.1093/eurheartj/ehad782)
Supplement: ehad782_Supplementary_Data [file ehad782_supplementary_data.zip › SupplementaryTable5.docx]

Supplementary Table 5 Model Performance on Subpopulations. When looking at performance within each subpopulation, there was no significant difference in the Composite label performance compared to the overall population performance.

|  |  | **SLVH** | | **DLV** | | **Composite SLVH/DLV** | |
| --- | --- | --- | --- | --- | --- | --- | --- |
|  |  | **AUROC** | **AUPRC** | **AUROC** | **AUPRC** | **AUROC** | **AUPRC** |
| **PM Excluded (n=3,178)** | **All CXRs** | .81 [.78, .83] | .23 [.18, .27] | .79 [.75, .83] | .16 [.09, .20] | .81 [.79, .84] | .36 [.30, .41] |
|  | **One CXR per patient** | .82 [.80, .84] | .23 [.20, .26] | .79 [.76, .82] | .13 [.08, .18] | .82 [.81, .84] | .32 [.28, .36] |
| **LT & HT Excluded**  **(n=2,796)** | **All CXRs** | .75 [.72, .77] | .24 [.19, .28] | .85 [.82, .88] | .41 [.34, .47] | .79 [.77, .81] | .44 [.39, .49] |
|  | **One CXR per patient** | .77 [.75, .78] | .25 [.23, .28] | .86 [.84, .88] | .37 [.33, .41] | .8 [.79, .81] | .42 [.39, .45] |
| **PM, LT, HT Excluded**  **(n=2,577)** | **All CXRs** | .76 [.73, .79] | .2 [.15, .23] | .78 [.74, .82] | .13 [.07, .17] | .77 [.75, .80] | .29 [.24, .33] |
|  | **One CXR per patient** | .75 [.73, .77] | .2 [.18, .22] | .81 [.78, .83] | .17 [.13, .20] | .77 [.76, .79] | .30 [.28, .32] |
