# Supplementary material for: Deep learning to detect left ventricular structural abnormalities in chest X-rays
Source: Eur Heart J. 2024 Mar 20;45(22):2002–12. doi: 10.1093/eurheartj/ehad782 (PMC11156488; doi:10.1093/eurheartj/ehad782)
Supplement: ehad782_Supplementary_Data [file ehad782_supplementary_data.zip › SupplementaryTable6.docx]

|  | **CUIMC Full** | **PM** | **LT** | **HT** | **CXR before First Echo** |
| --- | --- | --- | --- | --- | --- |
| **Number CXRs** | 71,589 | 5,069 | 9,176 | 7,126 | 583 |
| **Number Patients** | 24,689 | 2,026 | 563 | 789 | 438 |
| **Age** | 62.2 ± 16.2 | 68.2 ± 15.5 | 54.8 ± 14.5 | 58.4 ± 13.6 | 61.6 ± 16.0 |
| **Age Groups** |  |  |  |  |  |
| < 59 | 28,255 (39.5) | 1,398 (27.6) | 4,834 (52.7) | 3,291 (46.2) | 251 (43.1) |
| 60-69 | 19,384 (27.1) | 1,180 (23.3) | 3,217 (35.1) | 2,459 (34.5) | 142 (24.4) |
| 70-79 | 14,693 (20.5) | 1,252 (24.7) | 1,121 (12.2) | 1,256 (17.6) | 121 (20.8) |
| 80+ | 9,257 (12.9) | 1,239 (24.4) | 4 (0.0) | 120 (1.7) | 69 (11.8) |
| **Sex** |  |  |  |  |  |
| Female | 14,036 (56.8) | 909 (44.9) | 289 (51.2) | 236 (29.9) | 271 (61.9) |
| **Echo Measures** |  |  |  |  |  |
| IVS d 2D | 1.12  ± 0.27 | 1.24  ± 0.45 | 1.07  ± 0.18 | 1.20  ± 0.24 | 1.04  ± 0.25 |
| LVPW d 2D | 1.07  ± 0.22 | 1.15  ± 0.24 | 1.03  ± 0.18 | 1.15  ± 0.20 | 0.99  ± 0.21 |
| LV d 2D | 4.61  ± 0.68 | 5.06  ± 1.01 | 4.59  ± 0.52 | 4.82  ± 0.87 | 4.32  ± 0.50 |
| **Labels** |  |  |  |  |  |
| SLVH | 6,191 (8.6) | 799 (15.8) | 265 (2.9) | 720 (10.1) | 34 (5.8) |
| DLV | 4,343 (6.1) | 1,026 (20.2) | 207 (2.3) | 741 (10.4) | 4 (0.7) |
| Composite SLVH/DLV | 9,861 (13.8) | 1,663 (32.8) | 467 (5.1) | 1362 (19.1) | 38 (6.5) |

Supplementary Table 6 Subpopulation Characteristics. Data reported as mean ± SD or n (%). The only patient-level static characteristic is Sex. All other statistics are summarized on a per CXR-echocardiogram pair basis (including age as it may change across different CXRs for the same patient).
