# Supplementary material for: Deep learning to detect left ventricular structural abnormalities in chest X-rays
Source: Eur Heart J. 2024 Mar 20;45(22):2002–12. doi: 10.1093/eurheartj/ehad782 (PMC11156488; doi:10.1093/eurheartj/ehad782)
Supplement: ehad782_Supplementary_Data [file ehad782_supplementary_data.zip › SupplementaryTable7.docx]

|  | CUIMC Test | |
| --- | --- | --- |
|  | Count | Percentage |
| Number of Patients | 1,235 | -- |
| Number of CXRs | 3,667 | -- |
| Ethnicity |  |  |
| Non hispanic or Latino | 480 | 39% |
| Hispanic or Latino | 291 | 24% |
| Unknown/Declined/Other | 464 | 37% |
| Race |  |  |
| White | 460 | 37% |
| Black | 163 | 13% |
| Other combinations | 159 | 13% |
| Unknown/Declined | 413 | 34% |
| Asian | 28 | 2% |
| American Indian or Alaska Nation | 6 | <1% |
| Nat. Hawaiian / Other Pacific Island | 6 | <1% |
| Sex |  |  |
| Male | 528 | 43% |
| Female | 707 | 57% |
| Age* |  |  |
| 18-59 | 1428 | 39% |
| 60-69 | 1032 | 28% |
| 70-79 | 711 | 19% |
| 80+ | 496 | 14% |

Supplementary Table 7 Distribution of Race, Ethnicity, Age and Sex in CUIMC Test Set. We extracted the best available race and ethnicity information from our patient records for the test set. A significant complication of collecting this data at CUIMC is that patients can opt out of self-identification of race/ethnicity. While this is a patient-centered approach, it results in a significant portion of patients being labeled “unknown” or “declined”.

*Since age is a non-static characteristic, it is reported as a count of CXRs instead of a count of patients. All other statistics are counted on a per-patient basis.
