# Supplementary material for: Deep learning to detect left ventricular structural abnormalities in chest X-rays
Source: Eur Heart J. 2024 Mar 20;45(22):2002–12. doi: 10.1093/eurheartj/ehad782 (PMC11156488; doi:10.1093/eurheartj/ehad782)
Supplement: ehad782_Supplementary_Data [file ehad782_supplementary_data.zip › SupplementaryTable8.docx]

|  | **SLVH** | | **DLV** | | **Composite SLVH/DLV** | |
| --- | --- | --- | --- | --- | --- | --- |
|  | **Sensitivity** | **Specificity** | **Sensitivity** | **Specificity** | **Sensitivity** | **Specificity** |
| **PM Excluded (n=3,178)** | .86 [.81, .90] | .65 [.63, .66] | .87 [.81, .94] | .58 [.57, .59] | .87 [.84, .91] | .61 [.59, .63] |
| **LT & HT Excluded**  **(n=2,796)** | .75 [.70, .80] | .62 [.60, .64] | .82 [.77, .88] | .72 [.70, .74] | .74 [.70, .78] | .70 [.68, .71] |
| **PM, LT, HT Excluded**  **(n=2,577)** | .84 [.80, .89] | .55 [.53, .57] | .84 [.77, .91] | .61 [.59, .63] | .79 [.75, .84] | .61 [.59, .63] |
|  | **NPV** | **PPV** | **NPV** | **PPV** | **NPV** | **PPV** |
| **PM Excluded (n=3,178)** | .98 [.97, .98] | .16 [.14, .18] | .99 [.98, .99] | .06 [.05, .07] | .97 [.97, .98] | .21 [.18, .24] |
| **LT & HT Excluded**  **(n=2,796)** | .95 [.94 , .96] | .18 [.16, .20] | .98 [.97, 98] | .19 [.15, .22] | .93 [.92, .94] | .31 [.30, .34] |
| **PM, LT, HT Excluded**  **(n=2,577)** | .97 [.96, .98] | .16 [.14, .18] | .99 [.98, .99] | .07 [.06, .09] | .95 [.94, .96] | .22 [.19, .24] |

Supplementary Table 8 Model Performance on Subpopulations: Sensitivity, Specificity, NPV and PPV using Youden’s Threshold. We computed all four statistics using Youden’s threshold for each of the subpopulations to provide additional context for how the model performs at an individual point on the AU-ROC curve.
