# Supplementary figures and images for: Deep learning to detect left ventricular structural abnormalities in chest X-rays
Source: Eur Heart J. 2024 Mar 20;45(22):2002–12. doi: 10.1093/eurheartj/ehad782 (PMC11156488; doi:10.1093/eurheartj/ehad782)

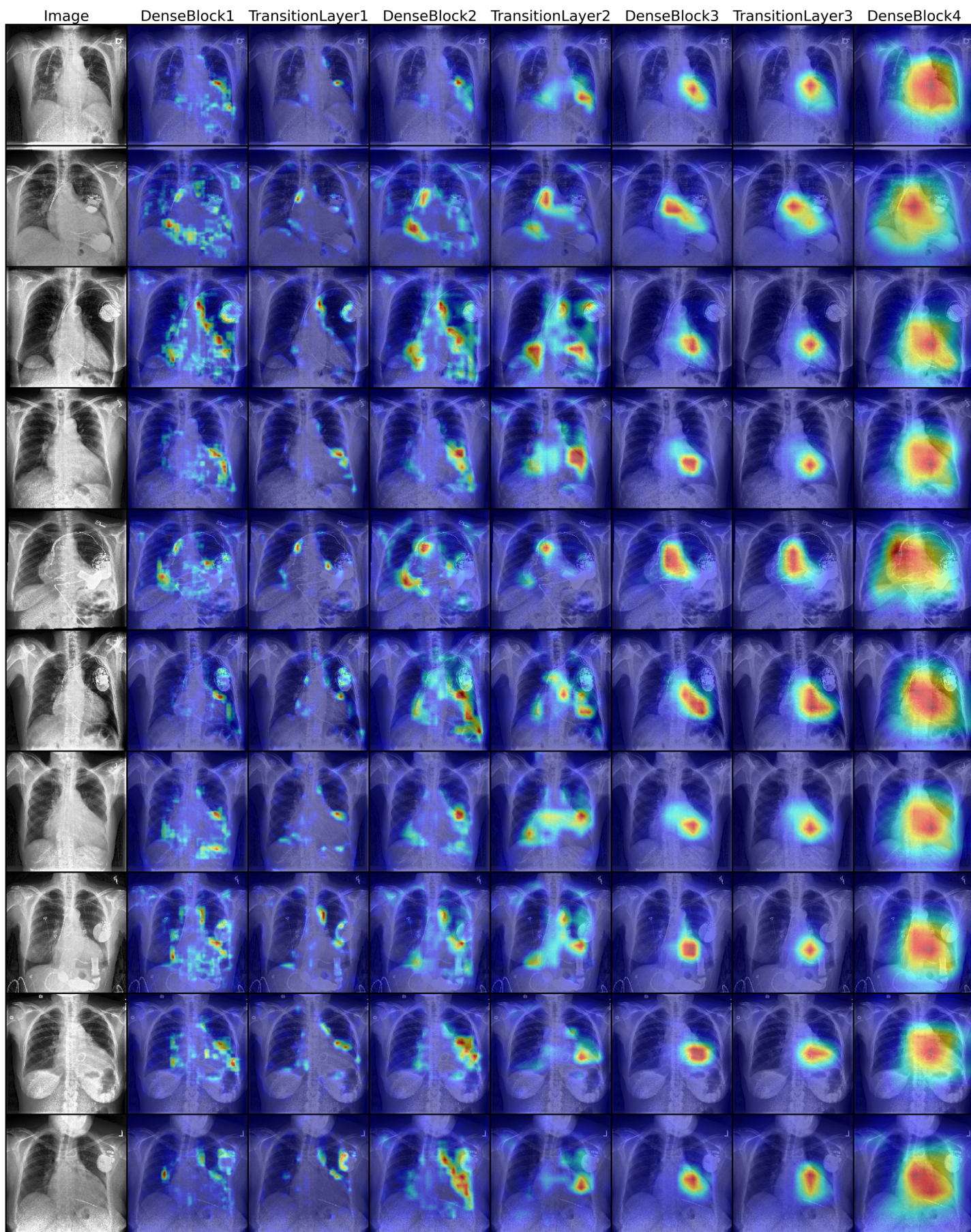

Supplement: ehad782_Supplementary_Data [file ehad782_supplementary_data.zip › SupplementaryFigure1.pdf]

**IVS d 2D Predicted vs True**

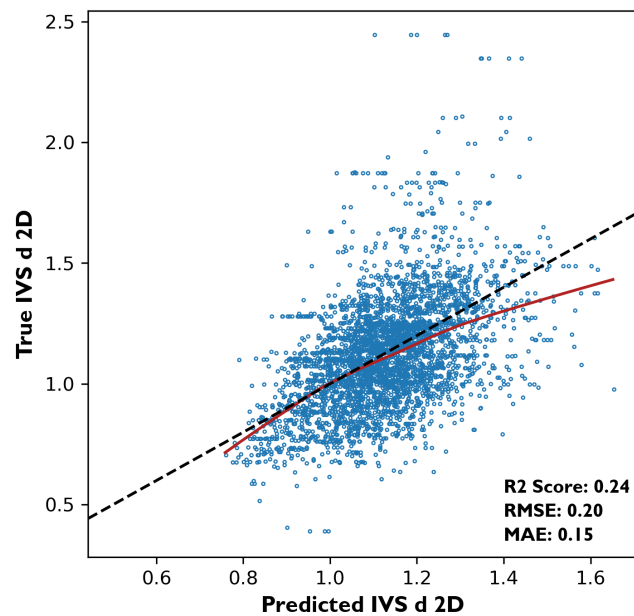

**LVPW d 2D Predicted vs True**

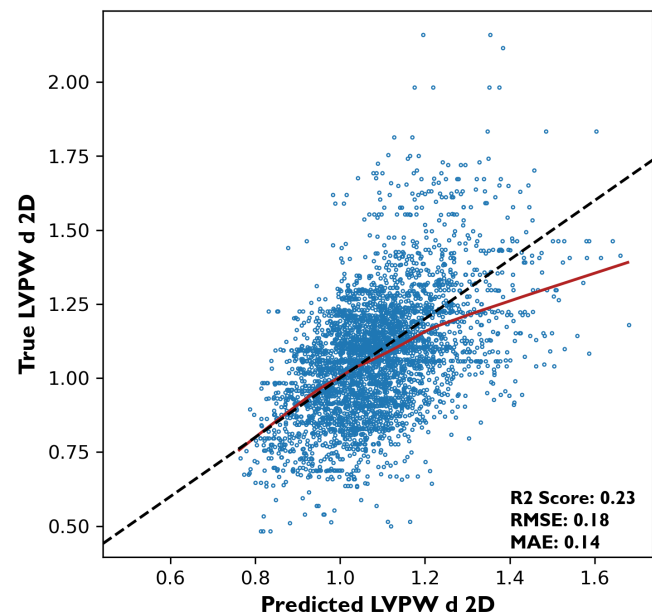

**LV d 2D Predicted vs True**

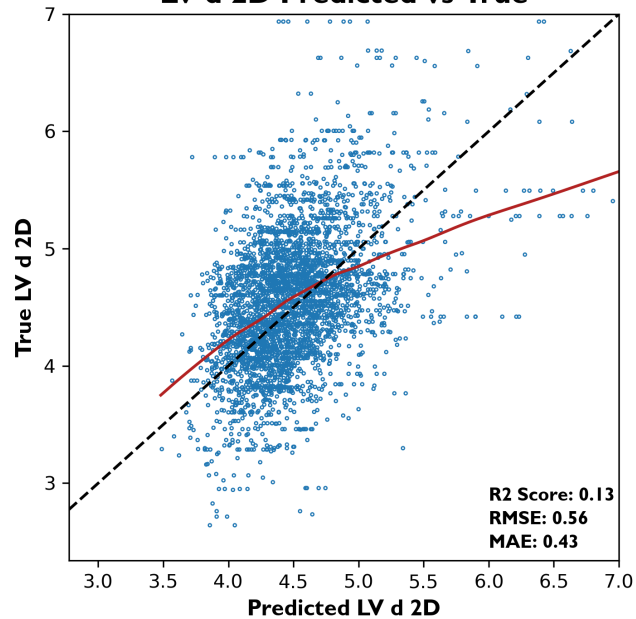

Supplement: ehad782_Supplementary_Data [file ehad782_supplementary_data.zip › SupplementaryFigure10Revised.pdf]

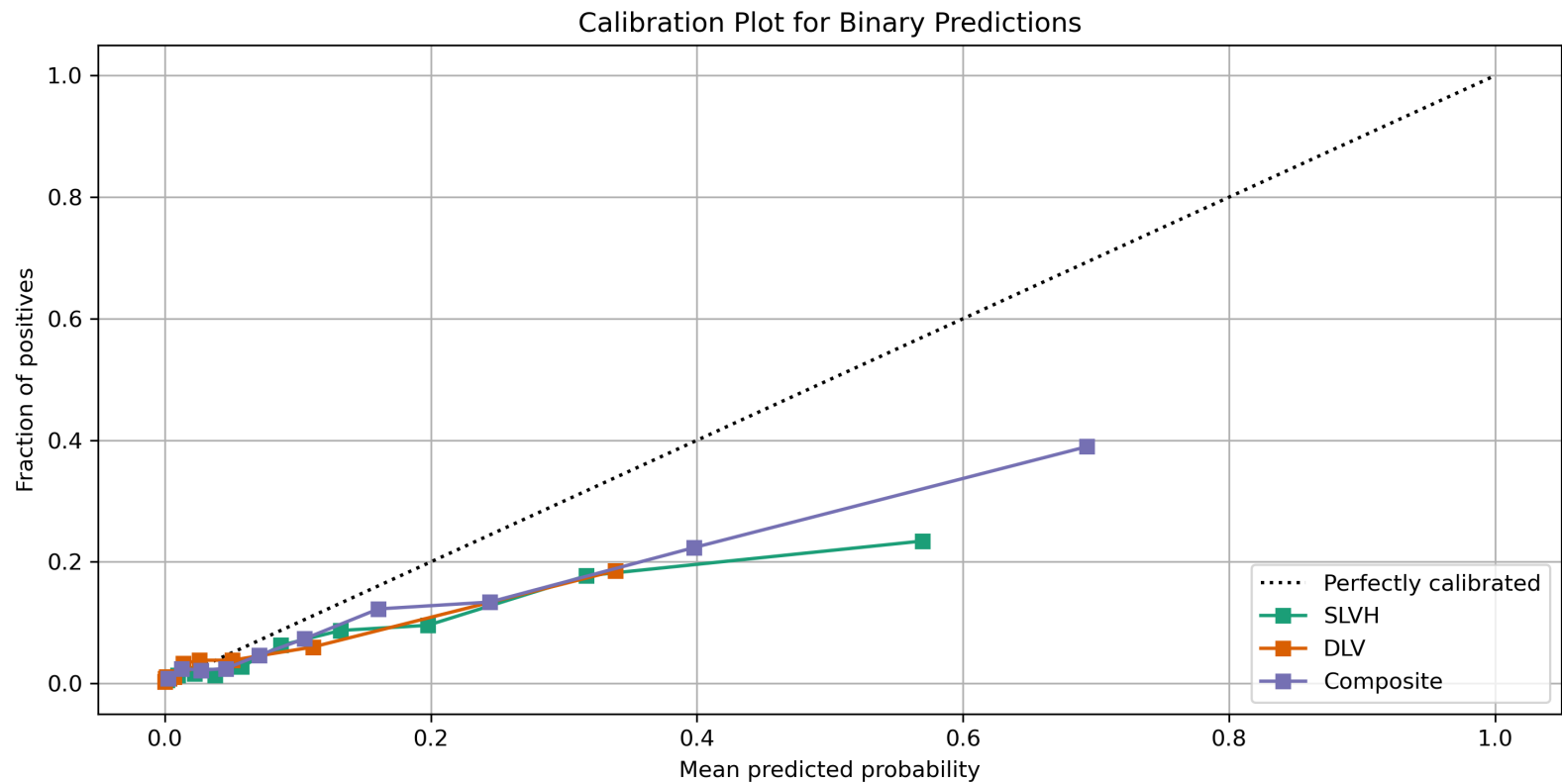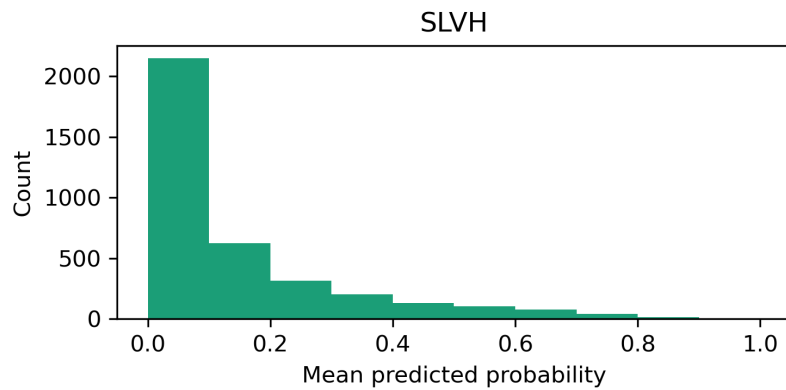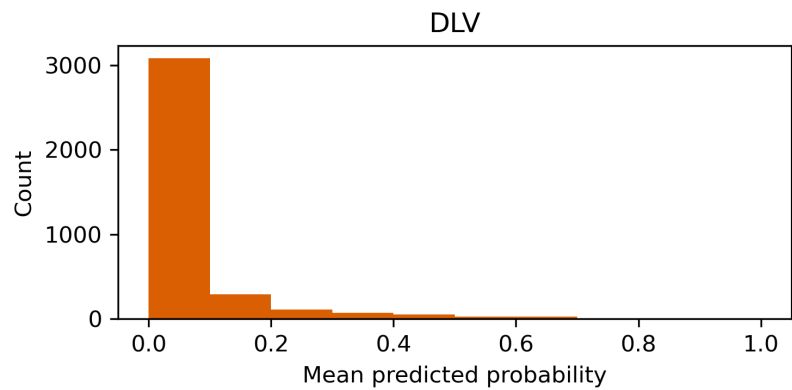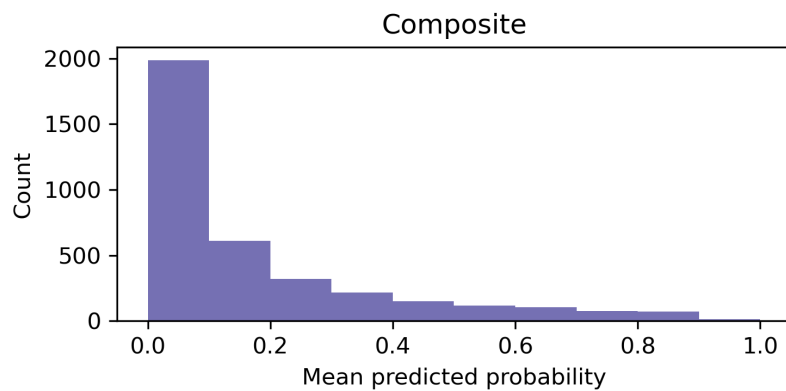

Supplement: ehad782_Supplementary_Data [file ehad782_supplementary_data.zip › SupplementaryFigure11.pdf]

Calibration Plot for Binary Predictions after Isotonic Regression

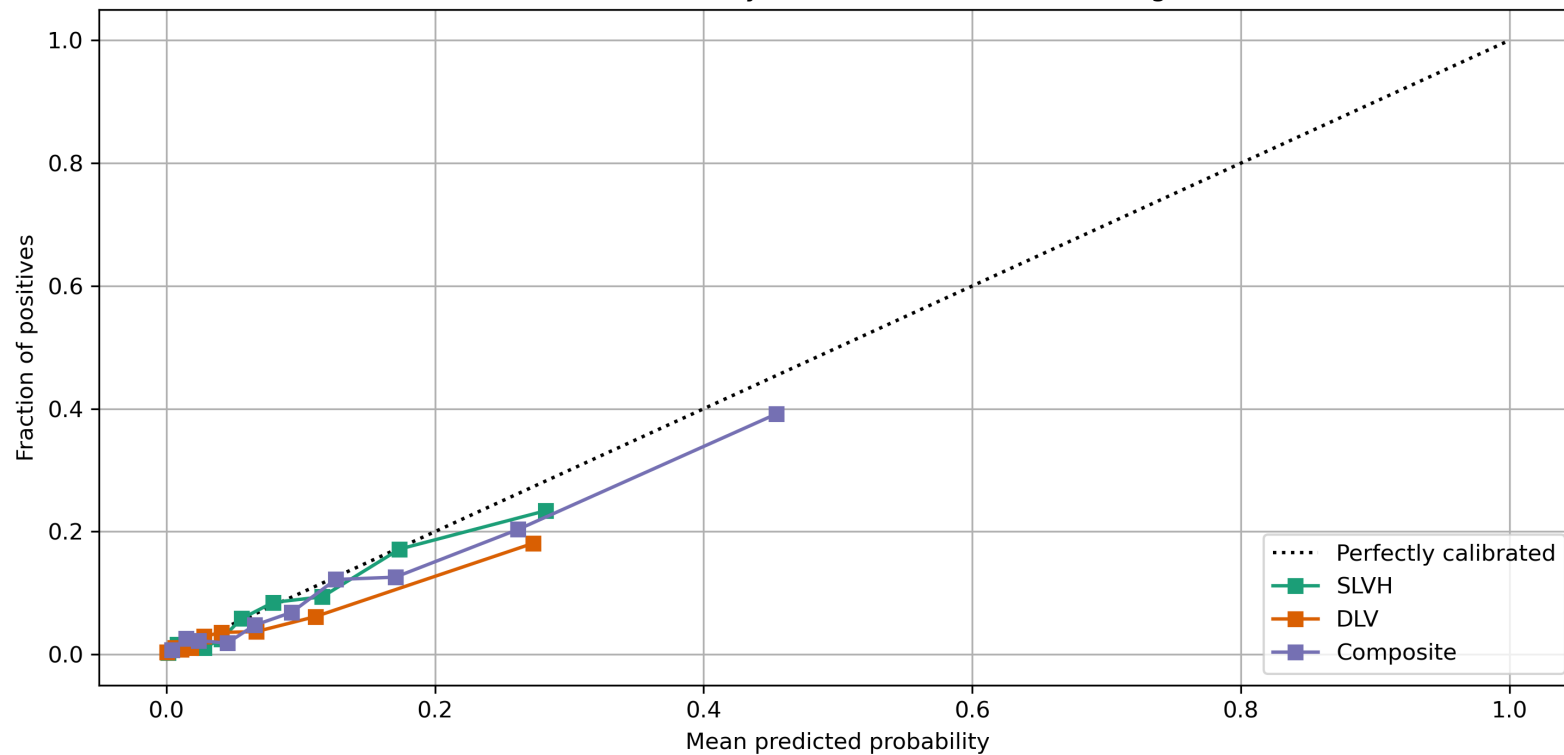

SLVH

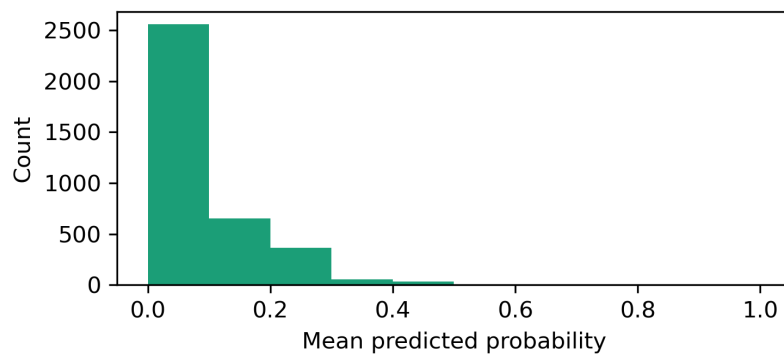

DLV

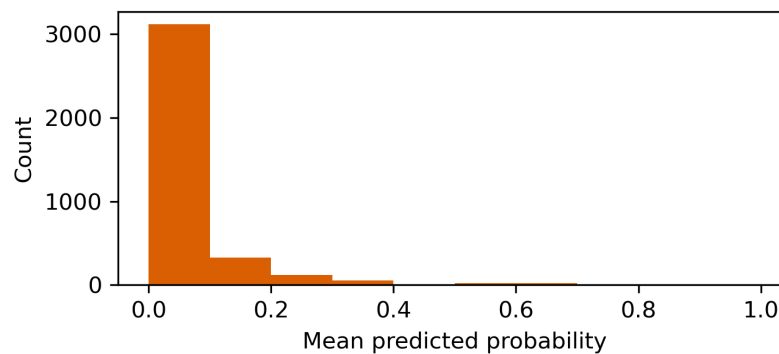

Composite

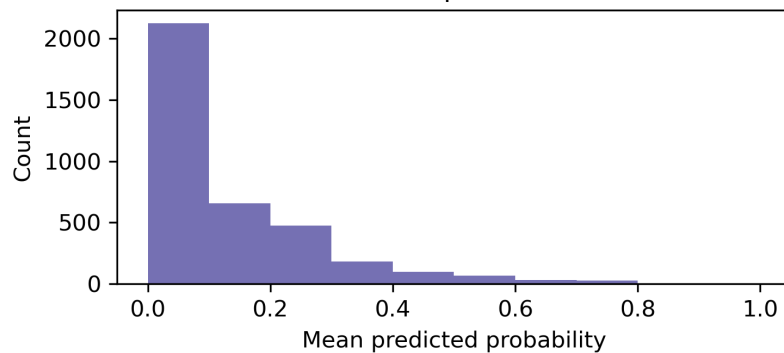

Supplement: ehad782_Supplementary_Data [file ehad782_supplementary_data.zip › SupplementaryFigure12.pdf]

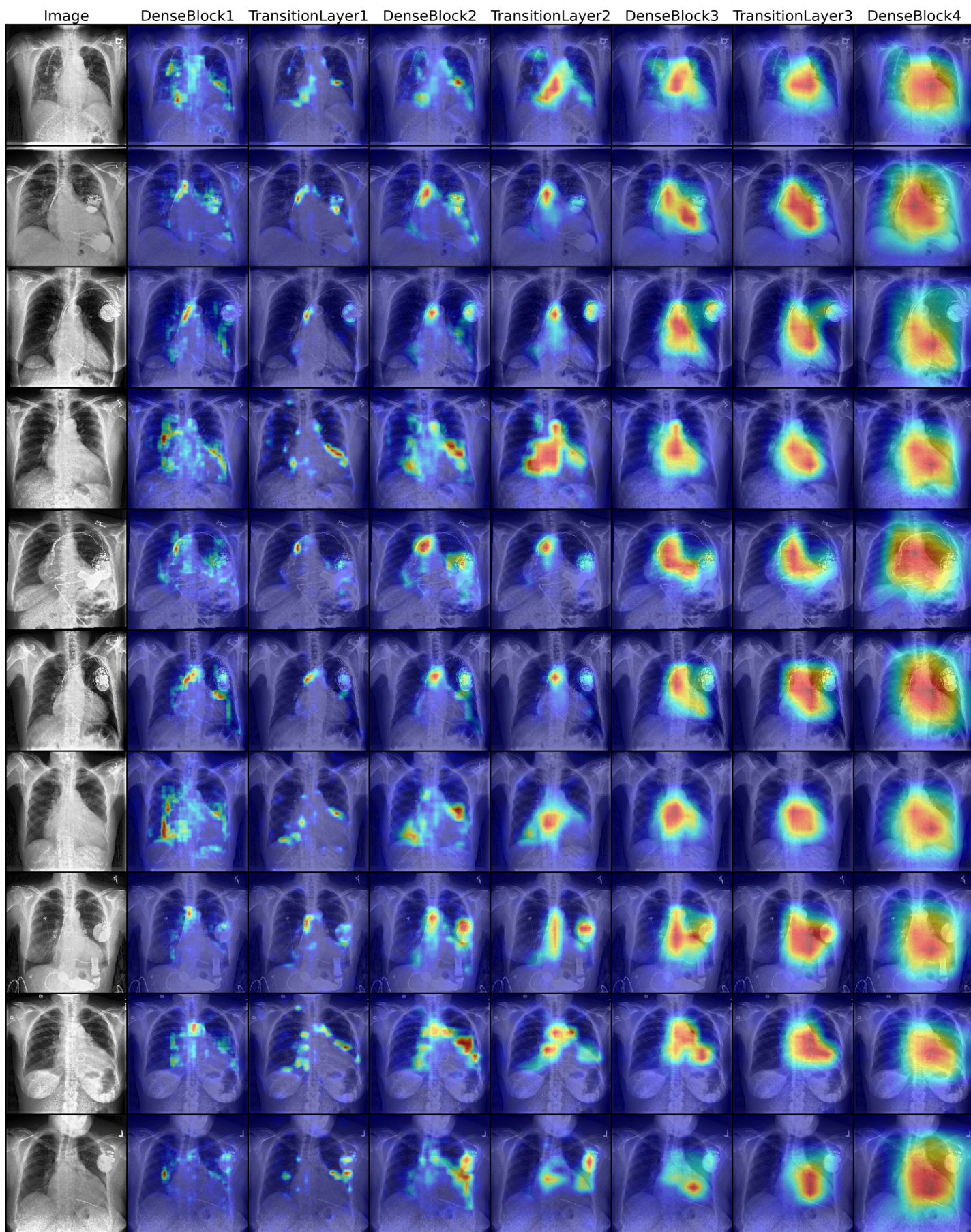

Supplement: ehad782_Supplementary_Data [file ehad782_supplementary_data.zip › SupplementaryFigure2.pdf]

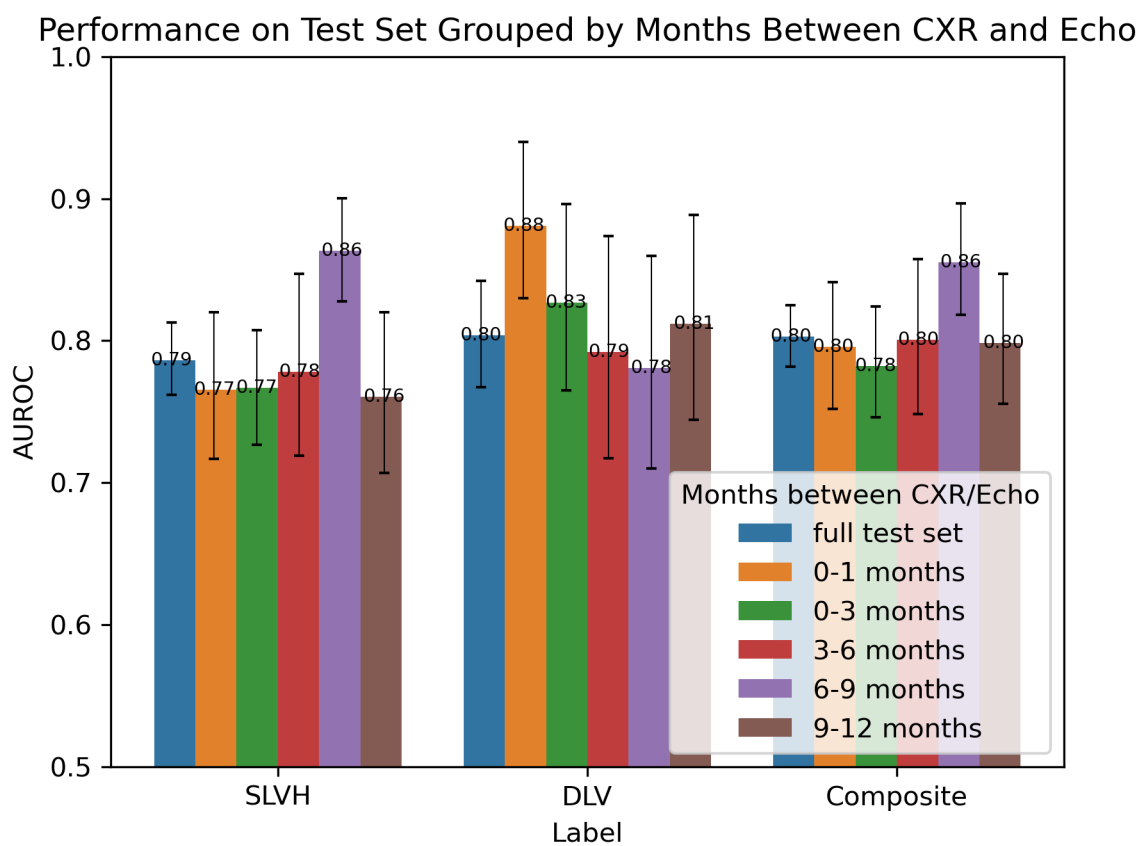

Supplement: ehad782_Supplementary_Data [file ehad782_supplementary_data.zip › SupplementaryFigure8Revised.pdf]

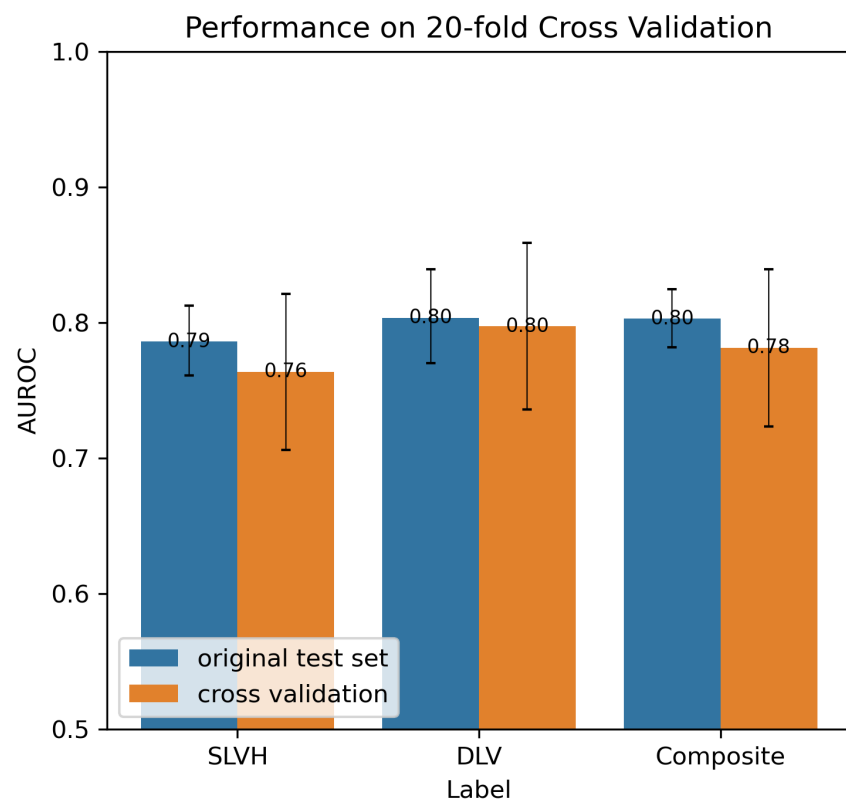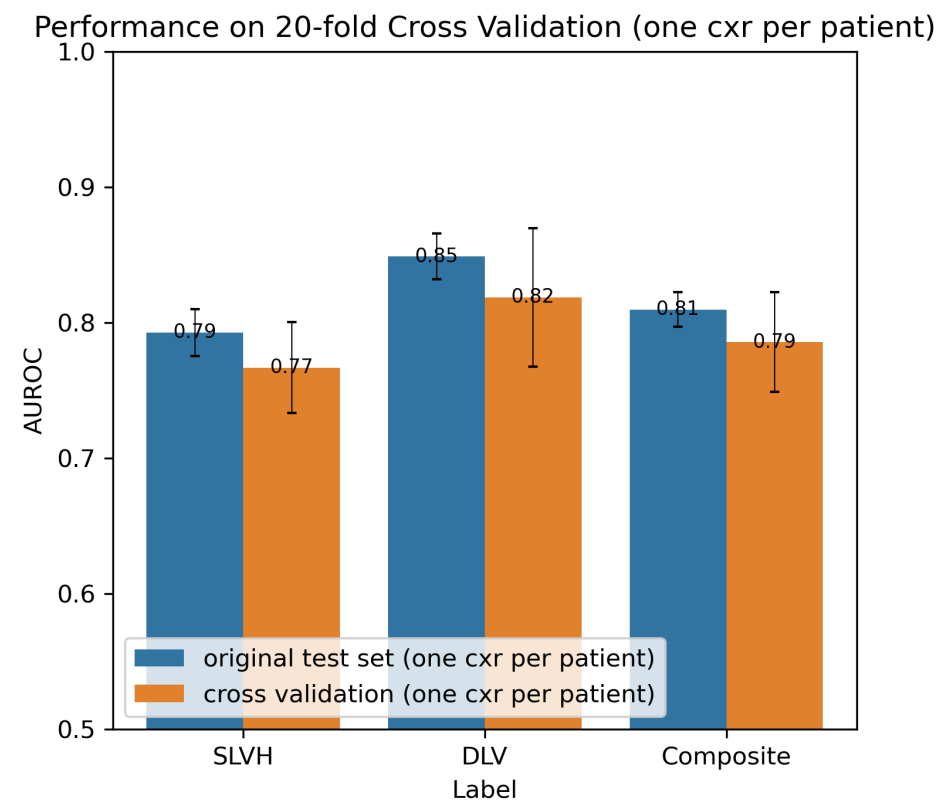

Supplement: ehad782_Supplementary_Data [file ehad782_supplementary_data.zip › SupplementaryFigure9Revised.pdf]
